# Hydrogenase Mimics in M_12_L_24_ Nanospheres to Control Overpotential and Activity in Proton‐Reduction Catalysis

**DOI:** 10.1002/anie.202008298

**Published:** 2020-08-17

**Authors:** Riccardo Zaffaroni, Nicole Orth, Ivana Ivanović‐Burmazović, Joost N. H. Reek

**Affiliations:** ^1^ Homogeneous, Supramolecular and Bio-Inspired Catalysis van't Hoff Institute for Molecular Sciences University of Amsterdam Science Park 904 1098 XH Amsterdam The Netherlands; ^2^ Department of Chemistry and Pharmacy Friedrich-Alexander-Universitaet Erlangen Egerlandstrasse 3 91058 Erlangen Germany

**Keywords:** catalysis, hydrogenases, proton reduction, substrate preorganization, supramolecular cages

## Abstract

Hydrogenase enzymes are excellent proton reduction catalysts and therefore provide clear blueprints for the development of nature‐inspired synthetic analogues. Mimicking their catalytic center is straightforward but mimicking the protein matrix around the active site and all its functions remains challenging. Synthetic models lack this precisely controlled second coordination sphere that provides substrate preorganization and catalyst stability and, as a result, their performances are far from those of the natural enzyme. In this contribution, we report a strategy to easily introduce a specific yet customizable second coordination sphere around synthetic hydrogenase models by encapsulation inside M_12_L_24_ cages and, at the same time, create a proton‐rich nano‐environment by co‐encapsulation of ammonium salts, effectively providing substrate preorganization and intermediates stabilization. We show that catalyst encapsulation in these nanocages reduces the catalytic overpotential for proton reduction by 250 mV as compared to the uncaged catalyst, while the proton‐rich nano‐environment created around the catalyst ensures that high catalytic rates are maintained.

## Graphical abstract

Hydrogenase mimic encapsulation in M_12_L_24_ nanospheres allows to create a special environment around synthetic mimics leading to a reduction of the proton reduction overpotential by 250 mV at the expense of catalytic rates while the creation of a proton‐rich environment leads to catalytic rates that are two orders of magnitude higher than without substrate preorganization.

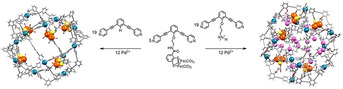

Hydrogenases are fascinating metalloenzymes that can reversibly convert protons into molecular hydrogen at high rates with virtually no overpotential.^1^ This reversible interconversion is of great interest in view of the transition from our current fossil fuel based society to one that is powered by renewable energy sources. As such, hydrogenase enzymes provide a powerful blueprint for the development of catalysts inspired by nature.^2^ Intensive studies on the iron‐iron hydrogenases showed their detailed operational mechanism and the key features that render these enzymes superb catalysts,^3^ revealing an important function for the internal proton relay,^4^ that is, the amine moiety in the azadithiolate bridge, and for the Fe_4_S_4_ cluster ligated to the proximal iron of the H‐cluster, which functions as electron reservoir. In parallel, many groups around the world made synthetic analogues of the active site at which the actual proton reduction takes place.^5^ Installment of proton relay moieties has been successfully achieved and demonstrated to improve the catalytic function of synthetic models.^6^ Less attention has been given to the redox‐active Fe_4_S_4_ cluster, nevertheless recent work on synthetic models with appended electron reservoirs demonstrated that such function also improves the catalytic properties.^7^ Interestingly, despite all efforts, up to now, there are no synthetic mimics that can perform the proton reduction reaction at low overpotential. This suggests that the protein environment, that is, the second coordination sphere around the active site, may play a more important role than initially anticipated.^8^ Recent experiments, in which synthetic mimics of the active site are installed in the inactive apo‐hydrogenase enzyme, show full competence enzymatic activity, hinting at the importance of the protein matrix or second coordination sphere around the H‐cluster.^9^ Introduction of a synthetic second coordination sphere around the hydrogenase mimics has been attempted using diverse strategies, chief among which liposomes,^10^ micelles,^11^ cyclodextrins,^12^ peptidic scaffolds^13^ and polymeric matrices^14^ yet information on catalysts activity and their overpotential remains rare.

Supramolecular cages represent an alternative strategy to control the second coordination sphere, and have proven successful in inducing enhanced activity and selectivity to the encapsulated catalysts.^15^ We recently showed that encapsulation of a single hydrogenase mimic into a tight supramolecular cage effectively provides a second coordination environment^16^ and this strategy resulted in lower catalytic overpotentials.^17^ In this work we report a self‐assembly strategy to install multiple mimics of the hydrogenase active site into very spacious M_12_L^I^_*n*_L^II^_24−*n*_ nano‐spheres based on mixtures of different ditopic bis(pyridyl) building blocks.^18^ With this strategy we can also create a specific proton‐rich nano‐environment by generating M_12_L^I^_*n*_L^II^_24−*n*_ nano‐spheres that contain ammonium salts as functional groups. We show that the hydrogenase models encapsulated in such cages are still electrocatalytically active for proton reduction. Most importantly, while we confirm that introducing a second coordination sphere around the synthetic catalyst is an effective strategy to lower the overpotential (about 250 mV) but at the expense of rate, we also demonstrate that proton preorganization leads to faster catalytic rates (about two orders of magnitude higher than without). This strategy allows to perform proton reduction catalysis at 350 mV overpotential which is 290 mV milder overpotentials as compared to our previously reported caged catalyst yet maintaining very similar catalytic rates.^17^

In order to create a nano‐environment able to effectively preorganize protons around di‐iron hydrogenase models, modified M_12_L^I^_*n*_L^II^_24−*n*_ Fujita‐type cages are employed. Such cages provide sufficiently large space within their cavity that can be easily decorated with various customized functional groups,18b that is, catalyst and acidic functions. The di‐iron catalyst functionalized building block **Fe_2_BB** features a short aliphatic linker that connects the ditopic bis(pyridyl) structure to a monocarboxylic acid benzenedithiolate di‐iron complex through an amide bond as shown in Figure 1. Ammonium groups are installed on different building blocks, **BBNH^+^**, in order to provide a proton rich local environment. This synthon features a short aliphatic chain terminated with a tertiary amine moiety. This base is about five orders of magnitude stronger than the pyridyl moieties present on the building block itself,^19^ allowing its selective protonation by just the addition of sub‐stoichiometric amounts of pyridinium hexafluorophosphate as confirmed by single crystal X‐ray diffraction (Figure S3). A third building block **BB** that doesn't contain functional groups at the endohedral position of the ditopic bis(pyridyl) building block is also prepared as shown in Figure 1.

**Figure 1:**
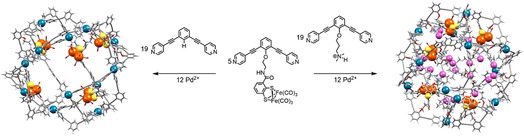
Schematic representation of the formation of cage [Pd_12_(**Fe_2_BB**)_5_(**BB**)_19_]^24+^ where the di‐iron catalyst is merely encapsulated within the cavity of the cage (left) and [Pd_12_(**Fe_2_BB**)_5_(**BBNH^+^**)_19_]^43+^ featuring di‐iron catalyst confined in a proton‐rich nano‐environment (right). The cage structures are optimized at molecular mechanics level (MMFF) and shown in wire‐style; carbon in grey, hydrogen in white, nitrogen in cyan, oxygen in red, metallic Pd corners as blue spheres. The di‐iron di‐sulfur cores of the hydrogenase mimics are represented as orange spheres (iron) and yellow spheres (sulfur). The acidic protons on **BBNH^+^** are represented as lilac spheres.

Cages were prepared using self‐assembly strategies as reported previously.15h, 15i, 20 Stirring a 5:19 ratio of **Fe_2_BB** and **BBNH^+^** (or **BB**) in the presence of a palladium source in MeCN at 60 °C overnight provided the two respective cages [Pd_12_(**Fe_2_BB**)_5_(**BBNH^+^**)_19_]^43+^ and [Pd_12_(**Fe_2_BB**)_5_(**BB**)_19_]^24+^.

The formation of the large nano‐cages is confirmed by ^1^H‐NMR, DOSY and HR‐CSI‐MS analysis. The ^1^H‐NMR shows a typical downfield shift of the pyridyl protons upon metal coordination (Figure S8 and S18). ^1^H‐DOSY‐NMR, a typical example shown in Figure 2, indicates the formation of a single diffusing species comprising signals belonging to both building blocks used and logD value of −9.3 m^2^ s^−1^, typical for these M_12_L_24_ spheres15h, 20 and diagnostic for the formation of the large well‐defined assembly. CSI‐MS data confirm the formation of the cage showing several signals belonging to cages of the type [Pd_12_(**Fe_2_BB**)_*n*_(**BBNH^+^**)_24−*n*_(PF_6_)_*x*_(CF_3_SO_3_)_*y*_]^*z*+^ (*n*=0–6) with different numbers of counter anions (*x* and *y*) and different charges *z* (details are found in SI). These experiments show that we can prepare systems in which the hydrogenase model is effectively encapsulated into a nano‐confined space with multiple mimics in one cage. For cage [Pd_12_(**Fe_2_BB**)_5_(**BBNH^+^**)_19_]^43+^ containing acidic protons, the di‐iron catalyst is in a proton‐rich nano‐environment where substrates are effectively preorganized around the catalyst within the cavity defined by the cage structure.

**Figure 2:**
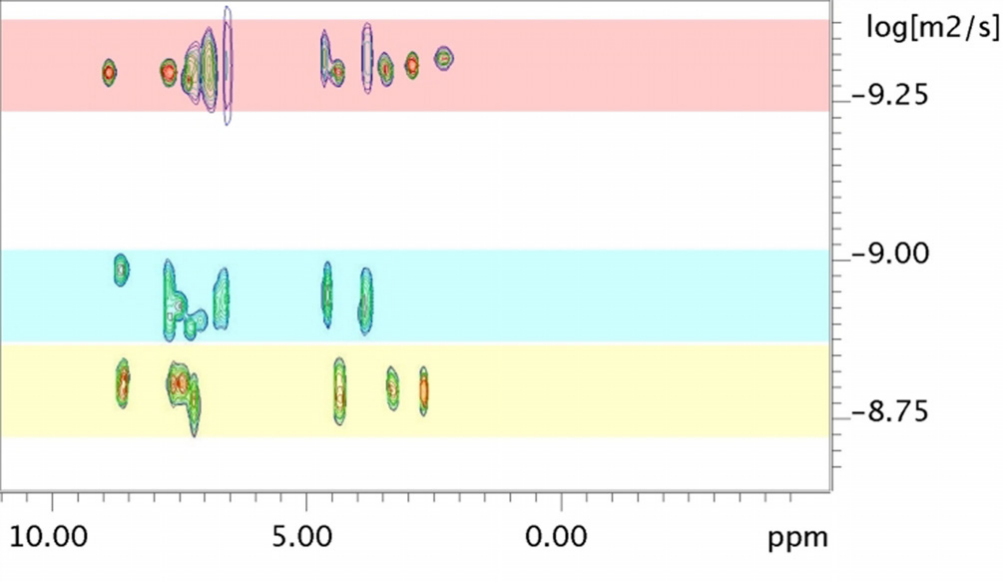
Overlay of ^1^H DOSY NMR in MeCN‐*d3* at 25 °C for a mixed cage of the type [Pd_12_(**Fe_2_BB**)_5_(**BBNH^+^**)_19_]^43+^ with log*D* of −9.3 m^2^ s^−1^ (top, red band). The **Fe_2_BB** shows a log*D* value of −8.9 m^2^ s^−1^ (middle, cyan band) and **BBNH^+^** shows a log*D* value of 8.8 m^2^ s^−1^ (bottom, yellow band).

Next the electrochemical proton reduction catalysis displayed by the caged catalysts was evaluated.^20^ For the cage type [Pd_12_(**Fe_2_BB**)_5_(**BB**)_19_]^24+^, where **BB** is the standard ditopic bis(pyridine) cage building block featuring a non‐acidic aromatic proton at the *endo* position, the voltammograms shows a reduction event around −1.3 V vs. Fc^0/+^ consistent with the reduction of the di‐iron moiety (Figure S24). Formation of the reduced catalyst is supported by IR‐spectroelectrochemical measurements showing its clear signature in the carbonyl region, indication that the caged di‐iron catalyst is stable under electrochemical conditions (Figure S35). Sequential additions of external weak acid, HNEt_3_PF_6_ not sufficiently strong to protonate the pyridyl groups or the non‐reduced iron‐iron bond, causes the appearance of a new peak at −1.7 V vs. Fc^0/+^ (Figure S25). This peak increases in intensity with the amount of acid added, in line with proton reduction catalysis at this potential. The modest increase in peak current intensity suggests that this catalytic process is rather slow. The external acid is able to diffuse into the cage cavity, but this may be relatively slow due to electrostatic repulsion between the positively charged acid and the positively charged cage shell. Interestingly, comparison of this catalytic peak potential to that obtained for the uncaged free **Fe_2_BB** reveals an anodic potential shift of about 230 mV towards more favorable potentials (Figure S26). The local environment around the catalyst lowers the overpotential for the catalytic proton reduction reaction probably due to stabilization of reduced reaction intermediates by the positive cage framework. So the positively charged cage results in more favorable overpotential for proton reduction catalysis and at the same time reduces the catalytic activity; k_cat_ is estimated by foot of the wave analysis^21^ to be 7.42×10^2^ mol^−1^ s^−1^ and TOF_max_ calculated to be 44 s^−1^ a decrease of two order of magnitude compared to the uncaged catalyst (*k*_cat_ **Fe_2_BB** 1.51×10^5^ mol^−1^ s^−1^).

We hypothesized that creation of a local acidic environment as designed for cage [Pd_12_(**Fe_2_BB**)_5_(**BBNH^+^**)_19_]^43+^ featuring acidic quaternary ammonium groups, would not show slow diffusion rate limitation because of the pre‐organization. When this cage is subjected to electrochemical analysis, the voltammogram displays a reduction event −1.7 vs. Fc^0/+^ as shown in Figure 3 and Figure S30. Addition of increasing equivalents of external acid to this same cage solution reveals a current increase of the peak at −1.7 vs. Fc^0/+^, in line with a proton reduction event. The catalytic rate constant observed for the proton preorganized‐encapsulated di‐iron catalyst is estimated to be in the order of 1.03×10^5^ mol^−1^ s^−1^; over two orders of magnitude faster than the nano‐confined catalyst in cage [Pd_12_(**Fe_2_BB**)_5_(**BB**)_19_]^24+^ without proton preorganization and approaching the catalytic rate of the uncaged **Fe_2_BB**, yet at lower overpotential. Whereas this proton pre‐organization is important for the hydrogenase mimics encapsulated in these large nanospheres, this is not observed for the caged catalyst {[Fe_4_(ZnL)_6_][Fe_2_(F_4_bdt)(PPy_3_)(CO_5_)]}^8+^ recently reported,^17^ which works at similar rates regardless of cage encapsulation. This smaller cage can accommodate only one catalyst and there is no space for co‐guests such as solvent molecules or electrolyte. The tight binding leads the catalyst to be in close contact with the cage walls, which may lead to stabilization of reaction intermediates (Figure S36). Because of the smaller size, the active site is closer to the bulk solution allowing a more rapid reaction with substrates. As such, substrate preorganization for this system is not needed for fast rates as substrate diffusion is not limiting catalysis. In contrast, the current M_12_L_24_ nano‐cage is much larger with a diameter of 5 nm and a volume over 30 times bigger. It can accommodate several catalysts as shown by CSI‐MS data and those are on average further away from the cage windows (Figure S36). As such, the two systems are rather different and so are the rates of substrate diffusion towards the caged catalyst. More detailed experiments are required to confirm these hypotheses.

**Figure 3:**
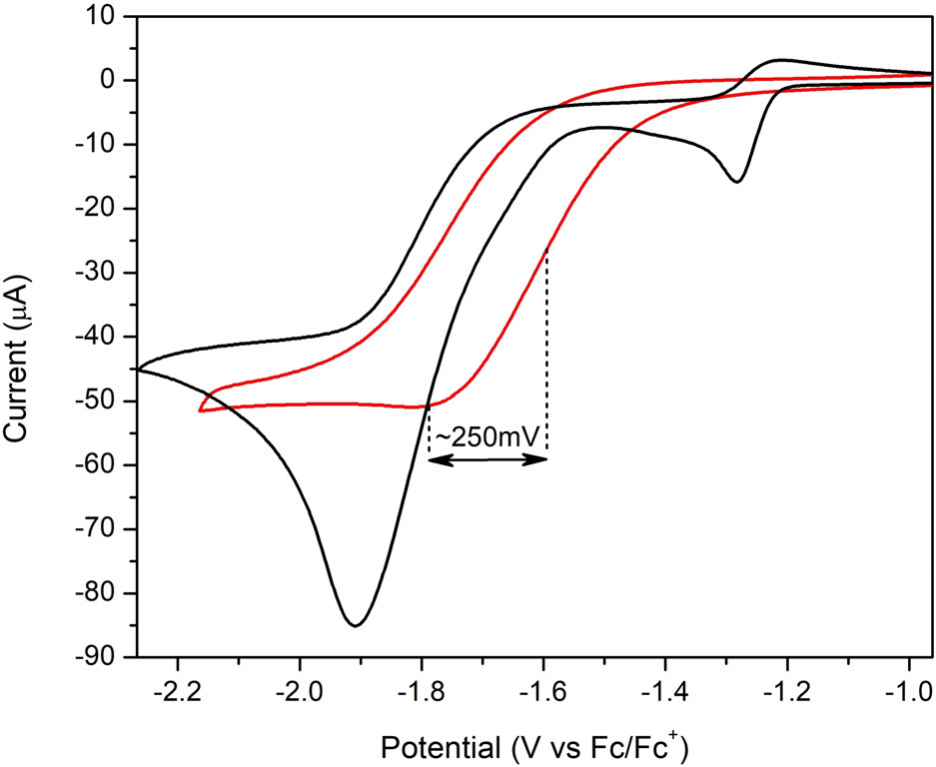
Cyclic voltammograms for cage [Pd_12_(**Fe_2_BB**)_5_(**BBNH^+^**)_19_]^43+^ in MeCN with 30 equivalents of external acid (red) and free **Fe_2_BB** in MeCN with 10 equivalents of external acid (black). For proton preorganized cage system, proton reduction takes place at about 250 mV milder potential. Scan speed 0.1 V s^−1^; **Fe_2_BB** 1 mm; cage [Pd_12_(**Fe_2_BB**)_5_(**BBNH^+^**)_19_]^43+^ 0.02 mm thus **Fe_2_BB** 0.1 mm due to solubility reasons (see FigureS28).

Importantly, the voltammograms obtained for the uncaged **Fe_2_BB** catalyst in the presence of acid and those obtained for the cage sample [Pd_12_(**Fe_2_BB**)_5_(**BBNH^+^**)_19_]^43+^, reveals that the catalytic half wave potential (*E* ${}_{1/2}$_cat_) is shifted anodically by 250 mV as shown in Figure 3. The cage effects are clear when plotting the properties for proton reduction catalysis in Tafel plots as shown in Figure 4. The encapsulation of the di‐iron catalyst leads to a reduction of the overpotential, for both cages investigated to only about 350 mV, which is among the lowest overpotential reported for this class of hydrogenase mimics. This suggests that the effect is unrelated to proton preorganization of the acidic moieties within the cage cavity but rather a cage effect possibly due to stabilization of negatively charged reaction intermediates by the positively charged cage framework. Instead, preorganization of proton substrates within the cavity of the supramolecular assembly has a beneficial effect as it allows for higher catalytic rates, stressing the importance of proton relays around the di‐iron moiety.

**Figure 4:**
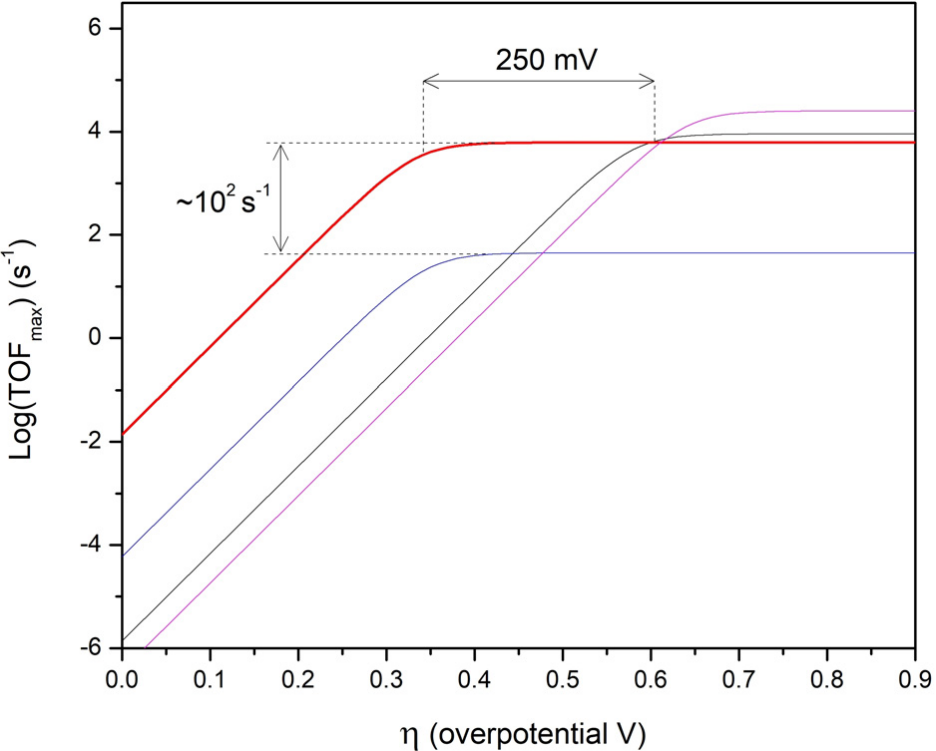
Tafel plot for free **Fe_2_BB** (black), cage [Pd12(**Fe_2_BB**)_5_(**BB**)_19_]^24+^ (blue), cage [Pd_12_(**Fe_2_BB**)_5_(**BBNH^+^**)_19_]^43+^ (red) and cage {[Fe_4_(ZnL)_6_][Fe_2_(F_4_bdt)(PPy_3_)(CO_5_)]}^8+^ 17 extrapolated at 30 mm external acid concentration, showing that catalyst encapsulation in proton‐rich environment leads to a drop in catalytic overpotential of 250 mV with respect to free diffusing **Fe_2_BB** while increasing the turnover frequency by two orders of magnitude compared to catalyst encapsulation in proton‐poor environment lacking substrate preorganization. Cage [Pd_12_(**Fe_2_BB**)_5_(**BBNH^+^**)_19_]^43+^ catalyses proton reduction at 290 mV milder overpotential as compared to previously reported cage [(Fe_4_(ZnL)_6_)(Fe_2_(F_4_bdt)(PPy_3_)(CO_5_)]^8+^ yet at similar rates.

The previously reported {[Fe_4_(ZnL)_6_][Fe_2_(F_4_bdt)(PPy_3_)(CO_5_)]}^8+^ system was based on a ligand template approach15l to encapsulation, which requires the catalyst to have a coordinated phosphine ligand, whereas the current system has an hexacarbonyl di‐iron derivative. Such a coordinated phosphine ligand increases the electron density at the di‐iron core and this typically results in faster catalytic rates but at the expense of higher overpotentials.6k As shown in Figure 4, {[Fe_4_(ZnL)_6_][Fe_2_(F_4_bdt)(PPy_3_)(CO_5_)]}^8+^ features the highest rate but also the largest overpotential while the combination of the more electron deficient hexacarbonyl catalyst and cage effect enables catalysis at 350 mV overpotential which represents a 290 mV reduction of catalytic overpotential as compared to the previously reported system. At the same time, substrate preorganization provided by the modified M_12_L_24_ cage allows for maintaining high proton reduction rates and in fact very comparable to those obtained by the electron richer monophoshine catalyst encapsulated in the smaller cage.

The strategy presented in this work allows to create a special environment around synthetic hydrogenase mimics, leading to improved performance in electrocatalytic proton reduction catalysis. The M_12_L_24_ cages provided a flexible platform to achieve a better understanding of second coordination sphere effects in catalysis and clear insights for future developments. Whereas we here demonstrate the effect of the cage and local concentration of protons (substrate), further modification to closely mimic the essential amino acid residues found around the structure of the natural H‐cluster may be possible. Introduction of synthetic mimics into nano‐environments such as the cavity of preferably precious‐metal‐free supramolecular cages decorated with such residues could further lower the overpotential of synthetic models, finally approaching enzymatic rates and efficiencies, a strategy that is currently explored in our laboratories.

## Conflict of interest

The authors declare no conflict of interest.

## Supporting information

As a service to our authors and readers, this journal provides supporting information supplied by the authors. Such materials are peer reviewed and may be re‐organized for online delivery, but are not copy‐edited or typeset. Technical support issues arising from supporting information (other than missing files) should be addressed to the authors.

Supplementary
