# Supplementary material for: Hydrogenase Mimics in M12L24 Nanospheres to Control Overpotential and Activity in Proton‐Reduction Catalysis
Source: Angew Chem Int Ed Engl. 2020 Aug 17;59(42):18485–9. doi: 10.1002/anie.202008298 (PMC7589440; doi:10.1002/anie.202008298)
Supplement: Supplementary file 1 — Supplementary [file ANIE-59-18485-s001.pdf]

Angewandte Chemie
Eine Zeitschrift der Gesellschaft Deutscher Chemiker

Supporting Information

Hydrogenase Mimics in $M_{12}L_{24}$ Nanospheres to Control Overpotential and Activity in Proton-Reduction Catalysis

*Riccardo Zaffaroni, Nicole Orth, Ivana Ivanović-Burmazović, and Joost N. H. Reek**

anie_202008298_sm_miscellaneous_information.pdf

Table of Contents

Materials and methods 2
Ligands synthesis and characterization 3
Building block **BBNH$^+$** 3
Building block **Fe_2BB** 6
Building block **BB** 8
Cages synthesis and characterization 10
Cage[Pd_{12}(**Fe_2BB**)$_5$(**BBNH$^+$**)$_{19}$]$^{43+}$ 10
Cage[Pd_{12}(**Fe_2BB**)$_5$(**BB**)$_{19}$]$^{24+}$ 21
Electrochemistry 23
Fe_2BB 23
Cage [Pd_{12}(**Fe_2BB**)$_5$(**BB**)$_{19}$]$^{24+}$ 26
Cage [Pd_{12}(**Fe_2BB**)$_5$(**BBNH$^+$**)$_{19}$]$^{43+}$ 29
Spectroelectrochemistry 32
References 35

Materials and methods

General procedures: all synthetic procedures were carried out under an argon or nitrogen atmosphere using standard Schlenk techniques. All commercially available chemicals were used as received without further purification. Solvents used for synthesis were dried, distilled and degassed with the most suitable method. Column chromatography was performed open to air using solvents as received.

Electrochemistry: Cyclic voltammetry was performed on 1 mM solution of analyte in MeCN (unless otherwise stated) using 0.1 M tetrabutylammonium hexafluorophosphate as supporting electrolyte. The voltammograms were recorded using a PG-STAT302N potentiostat at glassy carbon disk electrode (2 mm diameter). A platinum coil was used as auxiliary electrode and a leak free silver electrode (inner compartment 3 M KCl/Ag) as reference electrode
Spectroelectrochemistry was performed in an optically transparent thin layer Ottle cell with platinum working electrode, platinum auxiliary electrode and silver wire as reference electrode, containing 0.1 M tetrabutylammonium hexafluorophosphate as supporting electrolyte.

Mass analysis: high resolution mass spectra for all compounds were collected on an AccuTOF GC v 4g, JMS-T100G-CV mass spectrometer (JEOL, Japan).

Cryospray-ionization MS (CSI-MS): CSI-MS measurements were acquired on a UHR-ToF Bruker Daltonik (Bremen, Germany) maXis, and ESI-ToF MS with a resolution of at least 60.000 FWHM, coupled to a Bruker cryospray unit. Positive-ion detection mode with a source voltage between 4 kV and 5 kV were used. The flow rates were 180 uL/hour. Nitrogen drying gas was kept at -35 °C and the spray gas was kept at -40 °C. The machine was calibrated prior to every experiment via direct infusion of the Agilent ESI-ToF low concentration tuning mixture, which provided an m/z range of singly charged peaks up to 2700 Da in both ion modes.

X-ray Crystal Structure Determination: X-ray intensities were measured on a Bruker D8 Quest Eco diffractometer equipped with a Triumph monochromator (λ = 0.71073 Å) and a CMOS Photon 50 detector at a temperature of 150(2) K. Intensity data were integrated with the Bruker APEX2 software.[1] Absorption correction and scaling was performed with SADABS.[2] The structures were solved using intrinsic phasing with the program SHELXT.[1] Least-squares refinement was performed with SHELXL-2013[3] against F^2 of all reflections. Non-hydrogen atoms were refined with anisotropic displacement parameters. The H atoms were placed at calculated positions using the instructions AFIX 13, AFIX 43 or AFIX 137 with isotropic displacement parameters having values 1.2 or 1.5 times *U*eq of the attached C atoms.

Ligands synthesis and characterization

Building block BBNH+

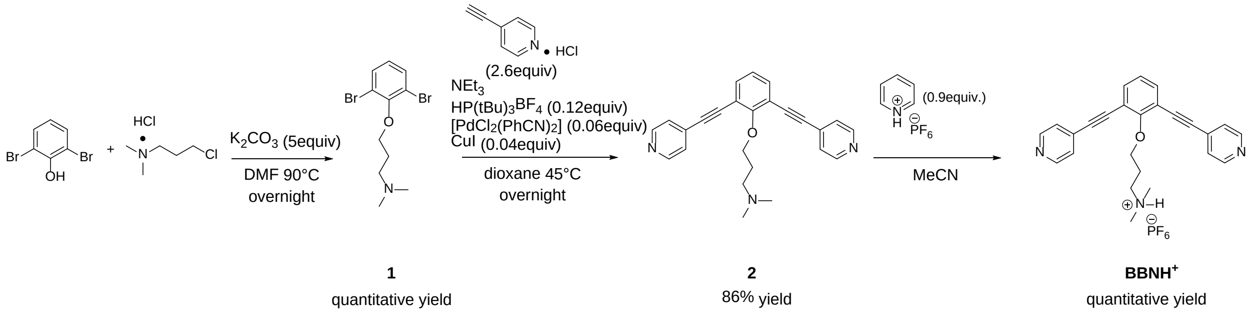

Scheme S1. Synthetic route for the preparation of **BBNH+**.

Synthesis of compound 1: A round bottom Schlenk flask was charged with 4.03 g of 2,6-di-bromophenol (1 equiv, 16 mmol) and 11 g of K_2CO_3 (5 equiv, 80 mmol). To this mixture, added 100 mL of dry and degassed DMF were added, followed by slow addition of 2.53 g of 3-chloro-N,N-dimethylpropan-1-amine hydrochloride (1 equiv, 16 mmol). The mixture was stirred overnight at 90 °C before being cooled to room temperature and the volatiles removed under reduced pressure. To the residue, 100 mL of water were added and the suspension extracted with dichloromethane (4x50 mL) then dried over $MgSO_4$ and the volatiles removed under vacuum. The crude mixture, whether needed can be purified by silica column chromatography with ethylacetate:triethylamine 99:1 to afford 5.38 g of **1** in quantitative yield. ^{1}H NMR (CD_2Cl_2, ppm) δ 7.55 (d, J = 8.0 Hz, 2H), 6.91 (t, J = 8.0 Hz, 1H), 4.08 (t, J = 6.6 Hz, 2H), 2.53 (t, J = 7.2 Hz, 2H), 2.26 (s, 6H), 2.05 (p, J = 6.8 Hz, 2H).

Synthesis of compound 2: A round bottom Schlenk flask was charged with **1**, 1.85 g (1 equiv, 5.50 mmol), 1.9 g of 4-ethynylpyridine hydrochloride (2.6 equiv, 14.3 mmol) and 42 mg of CuI (0.22 mmol). The flask was flushed with argon before a degassed mixture of 40 mL of dioxane and 10 mL of triethylamine was added. A separate Schlenk flask was charged with 126.6 mg of $Pd(PhCN)_2Cl_2$ (0.33 mmol) and 191.5 mg of $P(tBu)_3{\cdot}HBF_4$ (0.66 mmol). To this mixture, 5 mL of triethylamine and 10 mL of dioxane were added. This mixture was stirred for five minutes before it was transferred to the first flask by syringe. The mixture was stirred at 45 °C overnight then cooled to room temperature, quenched with water and extracted with ethyl acetate (4x50 mL). The organics were washed with water (2x50 mL) and brine (2x50 mL) then dried over $MgSO_4$ and the volatiles removed under vacuum. The residue was purified by silica column chromatography with ethyl acetate:trimethylamine 75:25. After the first impurities came out of the column 10% methanol was added to the eluent to elute the desired compound obtained as white solid, 2.1 g, 86% yield. ^{1}H NMR (CD_3CN, ppm) δ 8.77 – 8.54 (m, 4H), 7.64 (d, J = 7.7 Hz, 2H), 7.61 – 7.45 (m, 4H), 7.22 (t, J = 7.7 Hz, 1H), 4.43 (t, J = 6.3 Hz, 2H), 2.53 (t, J = 7.2 Hz, 2H), 2.14 (s, 6H), 2.03 (p, J = 6.6 Hz, 2H).

Synthesis BBNH+: A round bottom flask was charged with **2**, 1.0 g (1 equiv, 2.6 mmol) and 531 mg of pyridinium hexafluorophosphate (0.9 equiv, 2.34 mmol) The solids were dissolved in 20 mL of acetonitrile and stirred for 10 minutes before diethyl 50 mL of diethyl ether were added causing the precipitation of white solids. The solids were collected by filtration washed with hexanes and dried under vacuum to afford **BBNH+** in quantitative yield, 1.38 g. ^{1}H NMR (CD_3CN, ppm) δ 8.80 – 8.60 (m, 4H), 7.69 (d, J = 7.7 Hz, 2H), 7.61 – 7.43 (m, 4H), 7.29 (t, J = 7.7 Hz, 1H), 7.00 (br s, 1H), 4.44 (t, J = 5.7 Hz, 2H), 3.41 (t, J = 7.6 Hz, 2H), 2.81 (s, 6H), 2.27 (q, J = 7.1, 5.8 Hz, 2H). ^{13}C NMR (CD_3CN, ppm) δ 163.6, 153.4, 153.4, 138.4, 133.8, 128.7, 128.7, 128.2, 120.0, 94.5, 92.1, 75.0, 59.7, 46.6, 28.5. HR ESI(pos.)-MS (*m/z*) found: 382.1925 expected: 382.1919, $C_{25}H_{24}N_3O$.

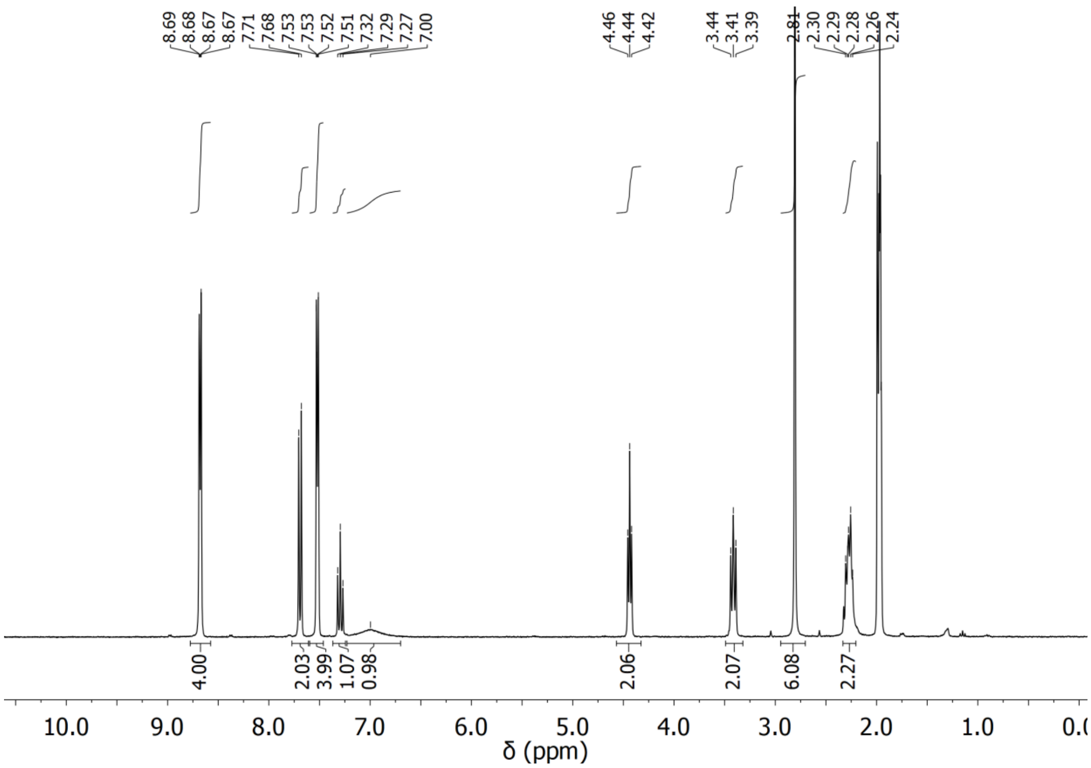

Figure S1. BBNH$^+$ building block, ^{1}H NMR in CD_3CN.

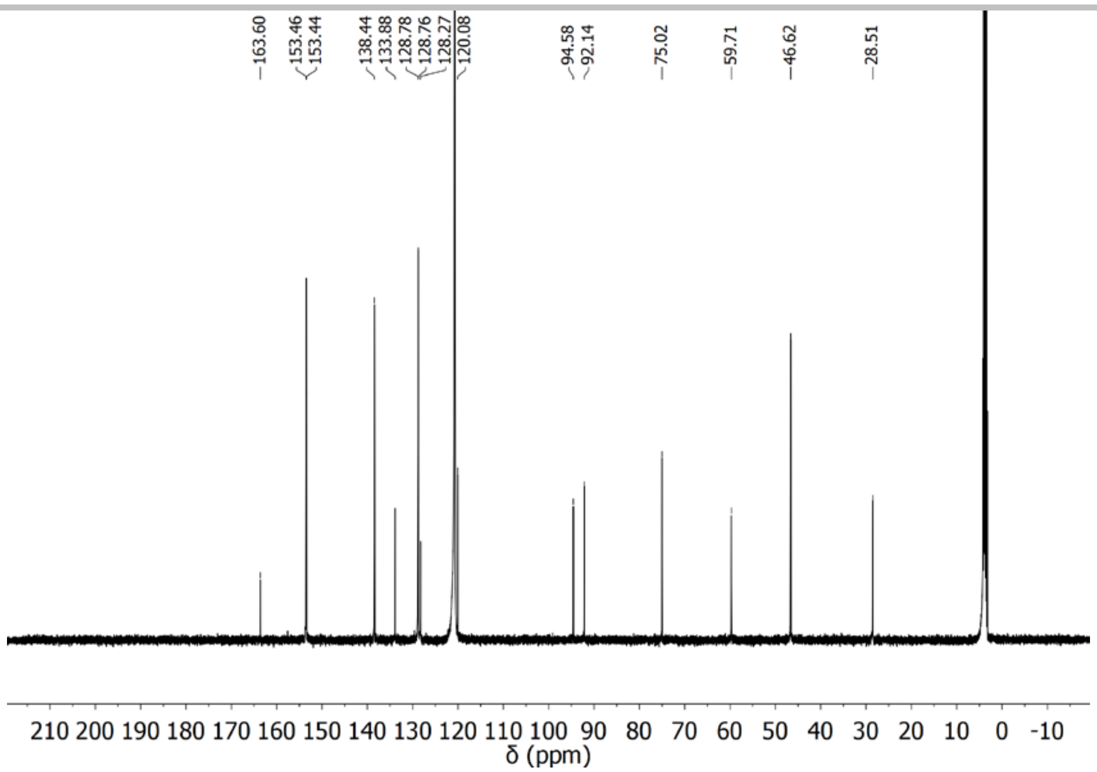

Figure S2. BBNH$^+$ building block, ^{13}C NMR in CD_3CN.

Crystallographic details

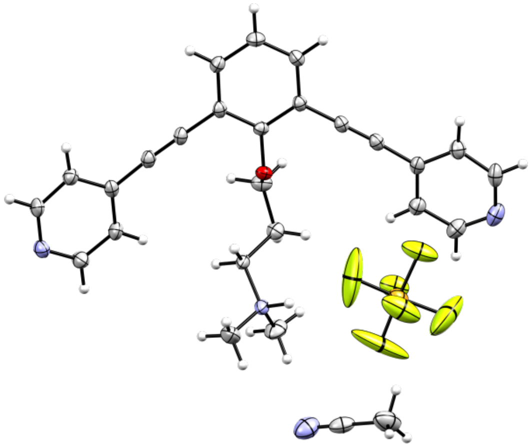

BBNHPF$_6$·MeCN: $C_{27}H_{27}F_6N_4OP$, Fw = 568.49, yellow plate, 0.37 × 0.26 × 0.16 mm, Monoclinic, *P*21/*n* (No: 14), a = 13.6743 (7), b = 14.1034 (8), c = 14.7769 (8) Å, β = 104.352 (3) °, V = 2760.8 (3) Å^3, Z = 4, Dx = 1.368 g/cm^3, μ = 0.17 mm^{-1}. 24875 reflections were measured up to a resolution of (sin θ/λ)max = 0.595 Å^{-1}. 4870 reflections were unique (R_{int} = 0.055), of which 3721 were observed [I>2σ(I)]. 355 parameters were refined with 0 restraints. R1/wR2 [I>2σ(I)]: 0.0613/0.1492 R1/wR2 [all refl.]: 0.0845/0.1694. S = 1.03. Residual electron density between −0.89 and 0.80 e/Å^3.

Figure S3. X-ray crystal structure of **BBNHPF$_6$·MeCN** building block. Ellipsoids are set at 50% probability. Carbon in gray, oxygen in red, nitrogen in blue, fluorine in yellow, phosphorous in orange and hydrogen in white. The presence of the acidic proton bound to the dimethyl amine group was established by the clear presence of residual electron density around the amino nitrogen in the difference Fourier map. Such residual electron density was not observed in the proximity of the pyridine nitrogen atoms.

Building block Fe_2BB

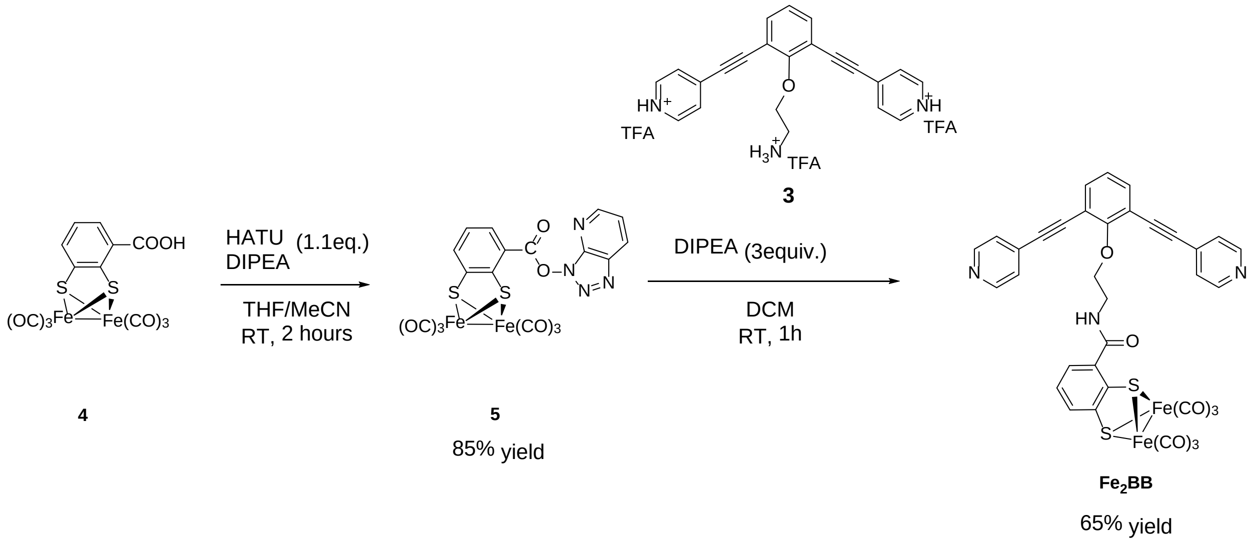

Scheme S2. *Synthetic route for the preparation of* ***Fe_2BB****.*

Preparation of the building block synthon **3** has recently been reported[4] while preparation of di-iron precursor **4** carried out according to literature procedure.[5]

Synthesis of compound 5: A round bottom Schlenk flask was charged with 250 mg of **4** (1 equiv, 0.59 mmol) and 249 mg of HATU (1.1 equiv, 0.65 mmol). The flask was flushed with argon before a degassed mixture of 20 mL of dry THF, 10 mL of dry acetonitrile and 0.5 mL of DIPEA was added. This mixture was stirred at room temperature for 2 hours before the volatiles were removed under vacuum. The residue was dissolved in dichloromethane and chromatographed over a DCM silica plug to afford the HATU-activated di-iron complex **5**, 295 mg, 85% yield, which was used immediately afterword.

Synthesis of Fe_2BB: A Schlenk flask was charged with compound **5** 295 mg (1 equiv, 0.51 mmol) and dissolved into 10 mL of DCM. A separate Schlenk flask was charged with building block **3** 274 mg (0.85 equiv, 0.43 mmol) and dissolved in a degassed mixture of 10 mL of DCM and 2 mL of DIPEA. The content of the first flask is added dropwise to the second flask and the mixture stirred at room temperature for an additional hour. The volatiles were removed under vacuum and the residue chromatographed over a silica column eluted with ethyl acetate:methanol 94:6 to afford **Fe_2BB** as mustard colored powder, 219 mg, 65% yield. ^{1}H NMR (CD_3CN, ppm) δ 8.65 – 8.55 (m, 4H), 7.66 (d, J = 7.8 Hz, 2H), 7.57 – 7.40 (m, 4H), 7.25 (d, J = 7.7 Hz, 1H), 7.24 (t, J = 7.8 Hz, 1H) 7.05 (br t, J = 5.0 Hz, 1H), 6.75 (d, J = 7.7 Hz, 1H), 6.60 (t, J = 7.6 Hz, 1H), 4.56 (t, J = 5.7 Hz, 2H), 3.82 (m, 2H). ^{13}C NMR (CD_2Cl_2, ppm) δ 207.3, 149.9, 134.9, 130.4, 129.4, 126.7, 126.0, 125.1, 124.1, 116.4, 89.0, 73.2, 40.3. FT-IR (MeCN, v) 2080, 2044, 2005 cm^{-1}. HR ESI(pos.)-MS (m/z) found: 785.9419 expected (m +H^+): 785.9392, (m +H^+) $C_{35}H_{20}Fe_2N_3O_8S_2$.

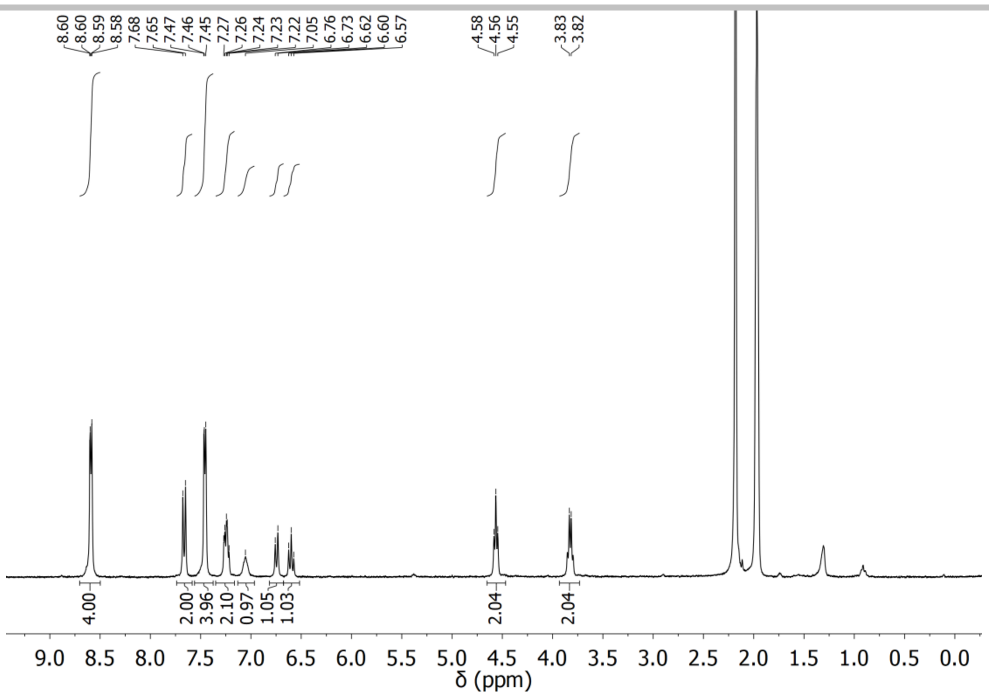

Figure S4. Fe_2BB building block, ^{1}H NMR in CD_3CN.

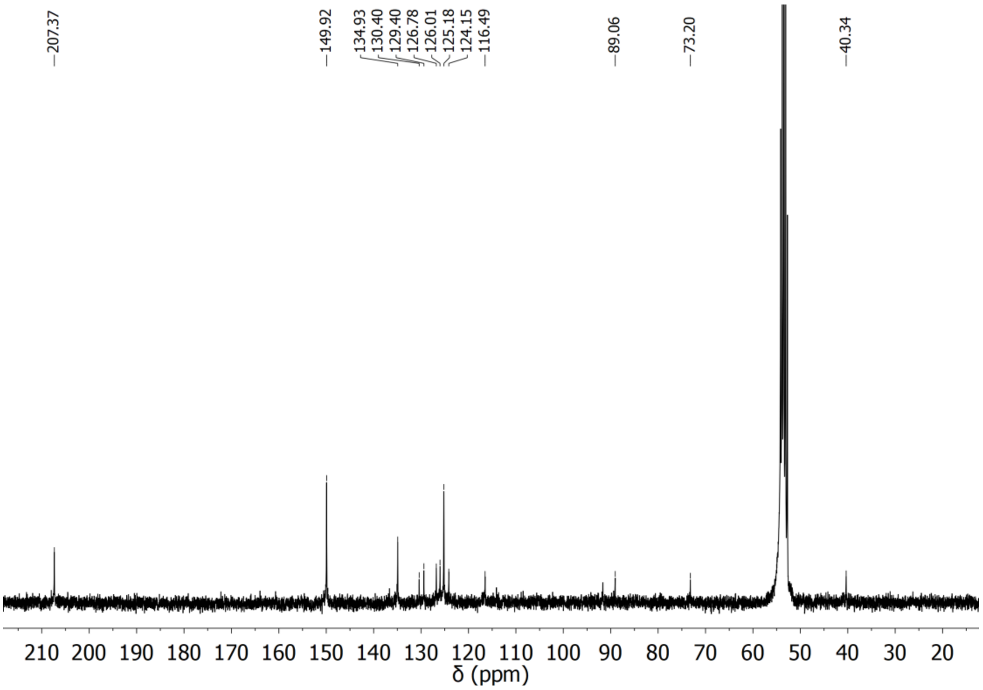

Figure S5. Fe_2BB building block, ^{13}C NMR in CD_2Cl_2.

Building block BB

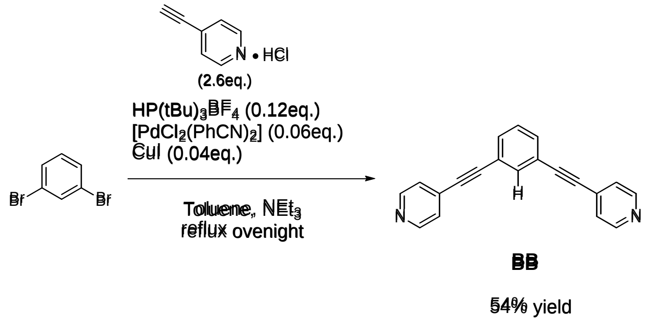

Scheme S3. Synthetic route for the preparation of **BB**.

Synthesis of BB: A round bottom Schlenk flask was charged with 1.62 g of 1,3-dibromobenzene (1 equiv, 6.9 mmol), 2.5 g of 4-ethynylpyridine hydrochloride (2.6 equiv, 17.9 mmol) and 52 mg of CuI (0.27 mmol). The flask was flushed with argon before a degassed mixture of 40 mL of toluene and 10 mL of triethylamine was added. A separate Schlenk flask was charged with 158 mg of Pd(PhCN)2Cl2 (0.41 mmol) and 240 mg of $P(tBu)_3{\cdot}HBF_4$ (0.82 mmol). To this mixture, 5 mL of triethylamine and 10 mL of toluene were added. This mixture was stirred for five minutes before it was transferred to the first flask by syringe. The mixture was stirred at 75 °C overnight then cooled to room temperature and filtered through a Celite plug. The organics were removed under vacuum and the residue dissolved in diethyl ether. The insoluble material was removed by filtration before diethyl ether was removed under vacuum. The residue is filtered through a plug of silica with a mixture of chloroform:methanol 96:4 to afford a mixture of mono-substituted derivative and **BB**. The solid material was suspended in 20 mL of ethyl acetate which solubilizes the mono-substituted compound along with some **BB**. Clean **BB** was obtained by filtration, 1.05 g, 54% yield. Nevertheless the mother liquors still contain high amounts of **BB** for which second crystallization could be attempted. ^{1}H NMR (CD_2Cl_2, ppm) δ 8.72 – 8.58 (m, 4H), 7.81 (s, 1H), 7.63 (dd, *J* = 7.8, 1.6 Hz, 2H), 7.48 (t, *J* = 7.8 Hz, 1H), 7.48 – 7.37 (m, 4H). ^{13}C NMR (CD_2Cl_2, ppm) δ 151.8, 136.8, 134.3, 132.6, 130.8, 127.3, 124.6, 94.2, 89.3. HR FD-MS (*m/z*) found: 280.0996 expected: 280.1000, $C_{20}H_{12}N_2$.

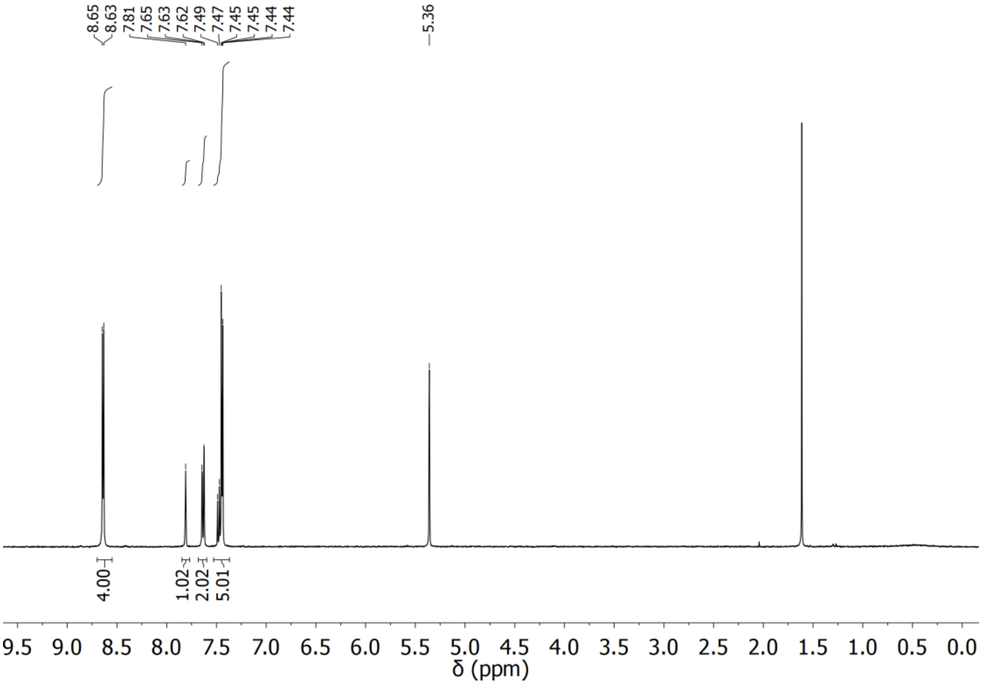

Figure S6. BB building block, ^{1}H NMR in CD_2Cl_2.

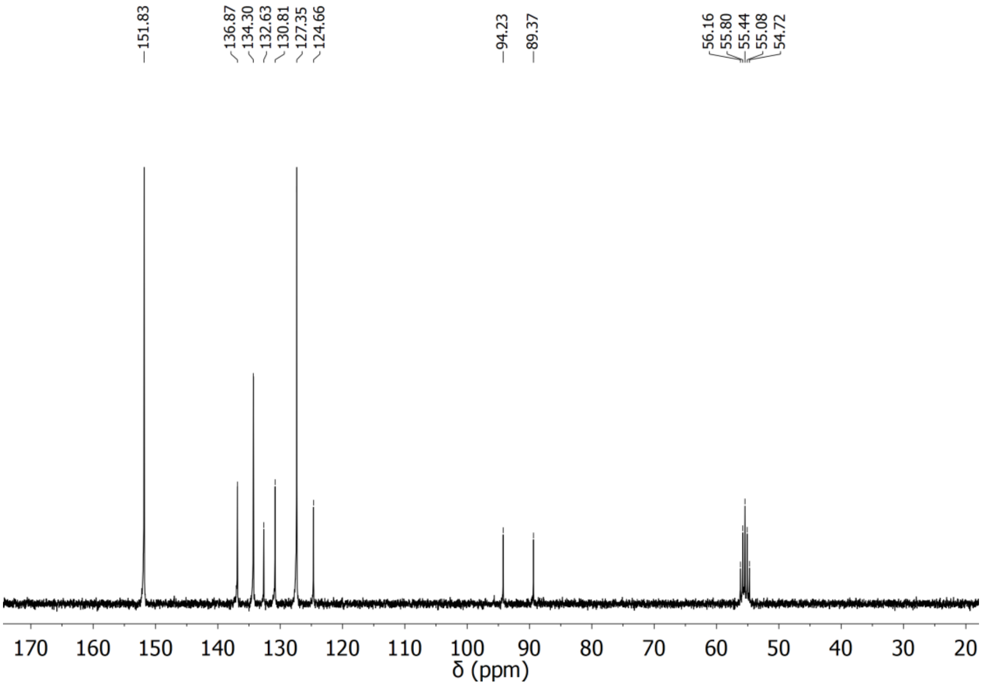

Figure S7. BB building block, ^{13}C NMR in CD_2Cl_2.

Cages synthesis and characterization

Cage[$Pd_{12}(Fe_2BB)_5(BBNH^+)_{19}]^{43+}$

Preparation: A Schlenk flask was charged with 3.93 mg (5 equiv, 5 µmol) of **Fe_2BB**, 10.02 mg (19eq, 19µmol) of **$BBNH^+$** and 6.72 mg (12 equiv, 12 µmol) of $[Pd(PF_6)_2(MeCN)_4]$. The flask was flushed with nitrogen before 5 ml of degassed CD_3CN were added. The resulting mixture was heated under N_2 at 60 °C overnight.

NMR Spectroscopy

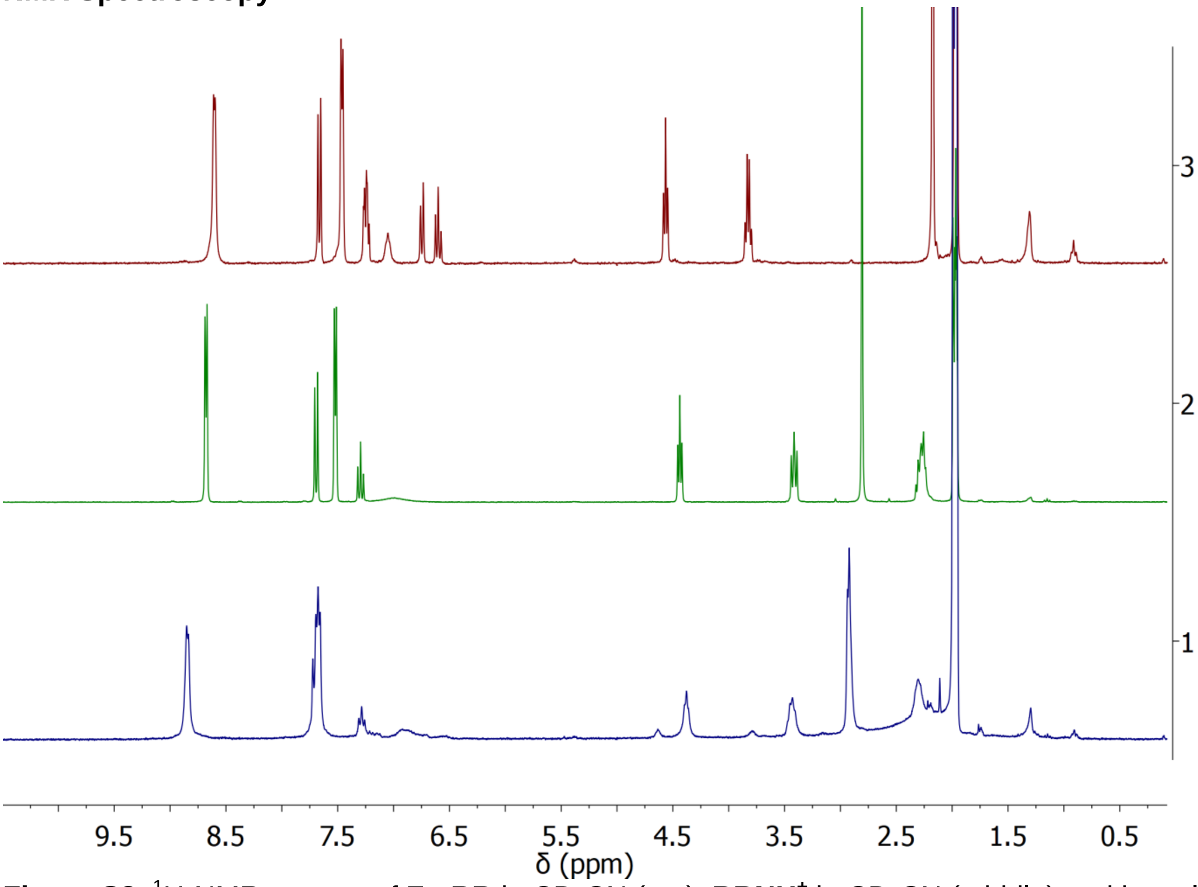

Figure S8. ^{1}H-NMR spectra of **Fe_2BB** in CD_3CN (top), **$BBNH^+$** in CD_3CN (middle) and its palladium cage $[Pd_{12}(\mathbf{Fe_2BB})_5(\mathbf{BBNH^+})_{19}]^{43+}$ in CD_3CN (bottom). Note shift of the pyridine peaks upon metal coordination.

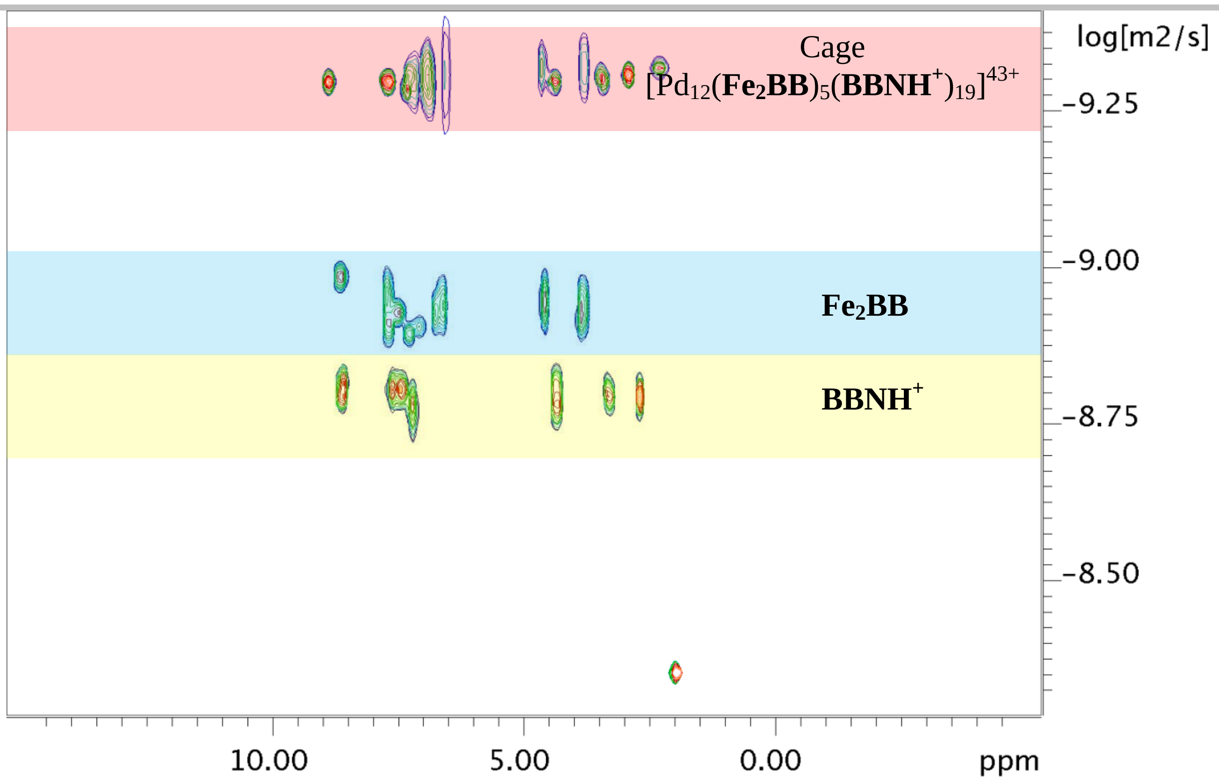

Figure S9. Overlay of ^{1}H DOSY NMR in MeCN-*d3* at 25 °C for a mixed cage of the type $[Pd_{12}(\mathbf{Fe_2BB})_5(\mathbf{BBNH^+})_{19}]^{43+}$ with logD of -9.3 m^2s^{-1} (top). This diffusing species contains the signals for both the ammonium functionalized building block and for the di-iron functionalized building block. The $\mathbf{Fe_2BB}$ shows a logD value of -8.9 m^2s^{-1} (middle) and $\mathbf{BBNH^+}$ shows a logD value of -8.8 m^2s^{-1}.

CSI-MS characterization

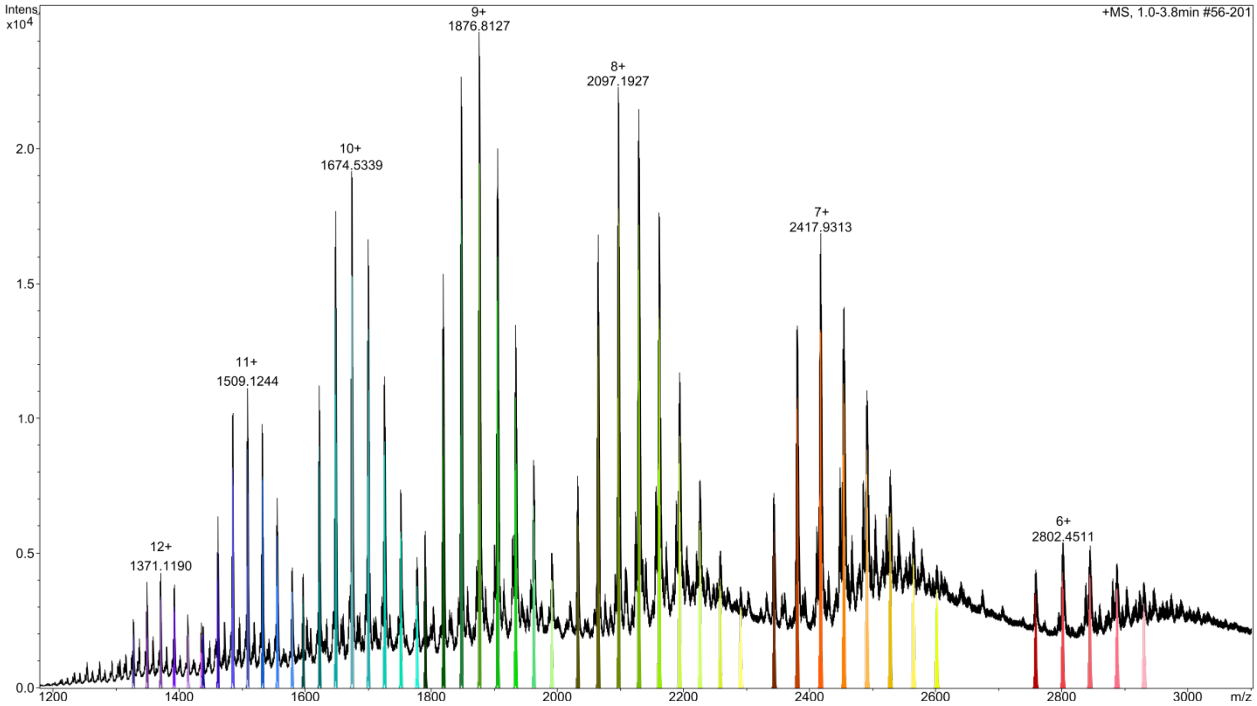

Figure S10. Full CSI-MS spectrum for cage sample $[Pd_{12}(\mathbf{Fe_2BB})_5(\mathbf{BBNH^+})_{19}]^{43+}$ in CD3CN (black) and expected species with of the type $[Pd_{12}(\mathbf{Fe_2BB})_n(\mathbf{BBNH^+})_{24-n}]$ (n=0-6) with different charges (colored overlays).

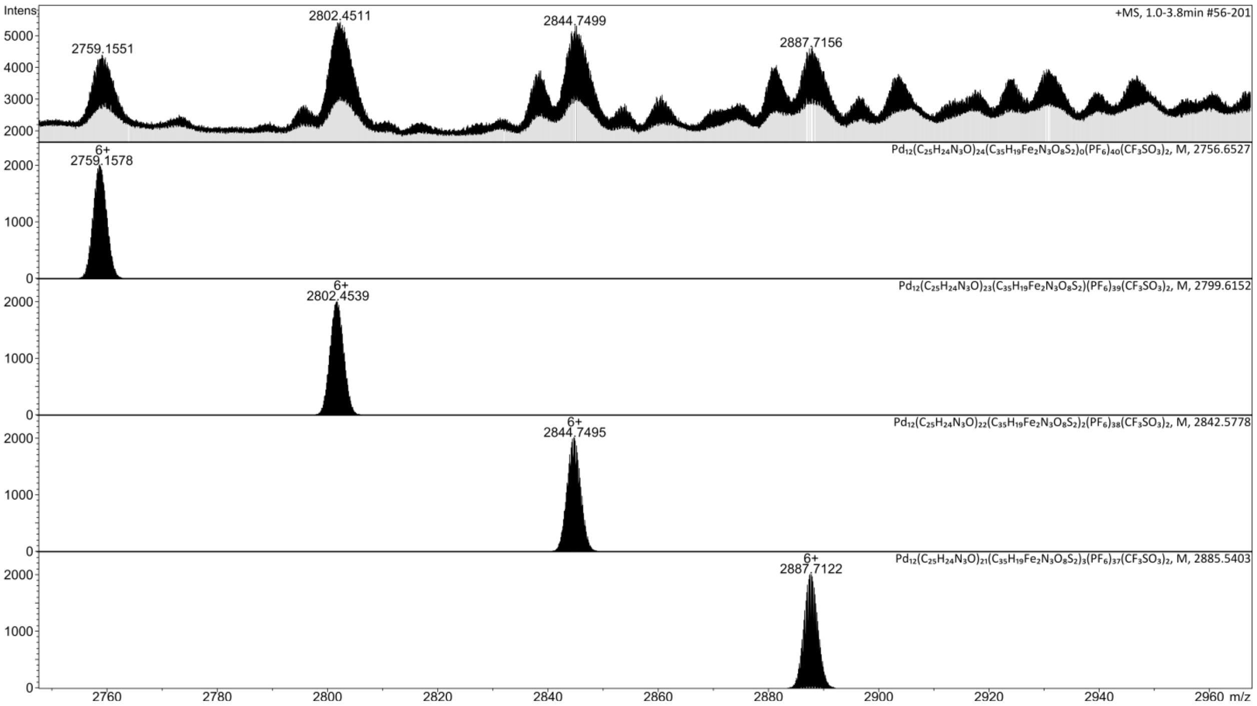

Figure S11. CSI-MS spectra for 6+ species observed for $[Pd_{12}(\mathbf{Fe_2BB})_5(\mathbf{BBNH^+})_{19}]^{43+}$ in CD3CN (top) and calculated species of the type $[Pd_{12}(\mathbf{Fe_2BB})_n(\mathbf{BBNH^+})_{24-n}(PF_6)_x(CF_3SO_3)_2]^{6+}$ (n=0-3; x=37-40). Addition of small amounts of CF_3SO_3H was found to be beneficial for the CSI-MS measurement.

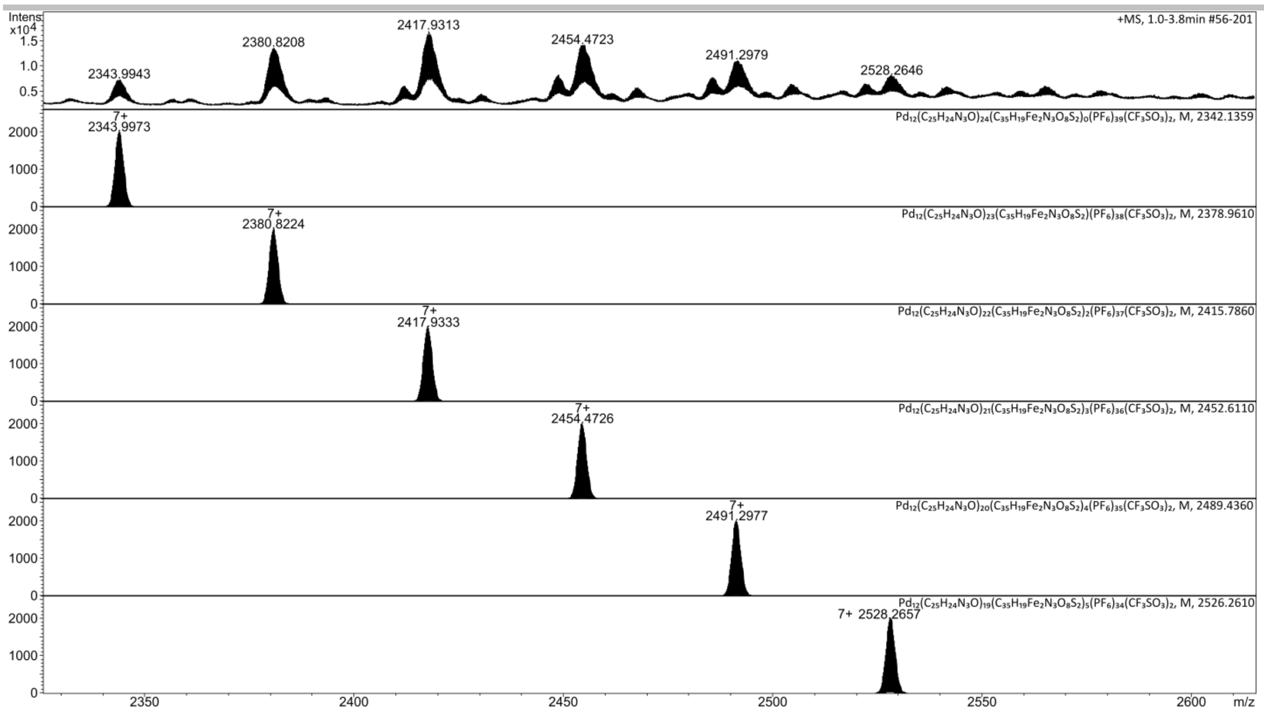

Figure S12. CSI-MS spectra for 7+ species observed for $[Pd_{12}(\mathbf{Fe_2BB})_5(\mathbf{BBNH^+})_{19}]^{43+}$ in CD3CN (top) and calculated species of the type $[Pd_{12}(\mathbf{Fe_2BB})_n(\mathbf{BBNH^+})_{24-n}(PF_6)_x(CF_3SO_3)_2]^{7+}$ (n=0-5; x=34-39). Addition of small amounts of CF_3SO_3H was found to be beneficial for the CSI-MS measurement.

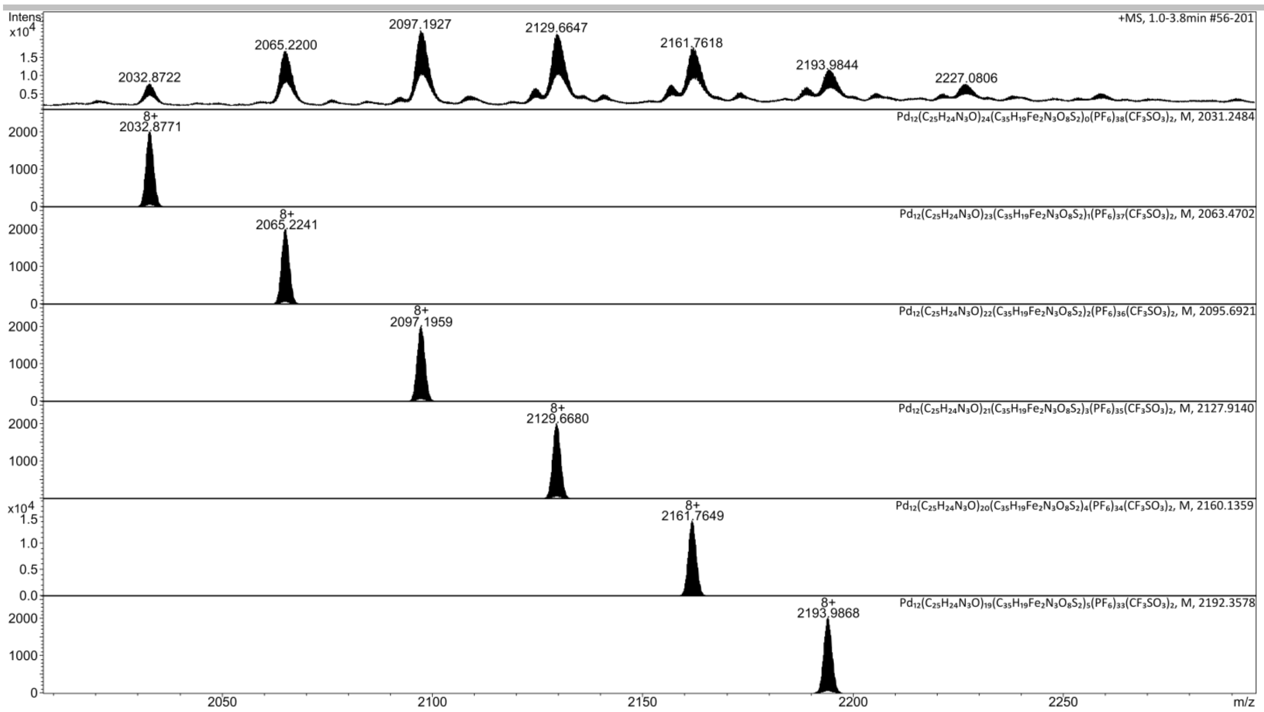

Figure S13. CSI-MS spectra for 8+ species observed for $[Pd_{12}(\mathbf{Fe_2BB})_5(\mathbf{BBNH^+})_{19}]^{43+}$ in CD3CN (top) and calculated species of the type $[Pd_{12}(\mathbf{Fe_2BB})_n(\mathbf{BBNH^+})_{24-n}(PF_6)_x(CF_3SO_3)_2]^{8+}$ (n=0-5; x=33-38). Addition of small amounts of CF_3SO_3H was found to be beneficial for the CSI-MS measurement.

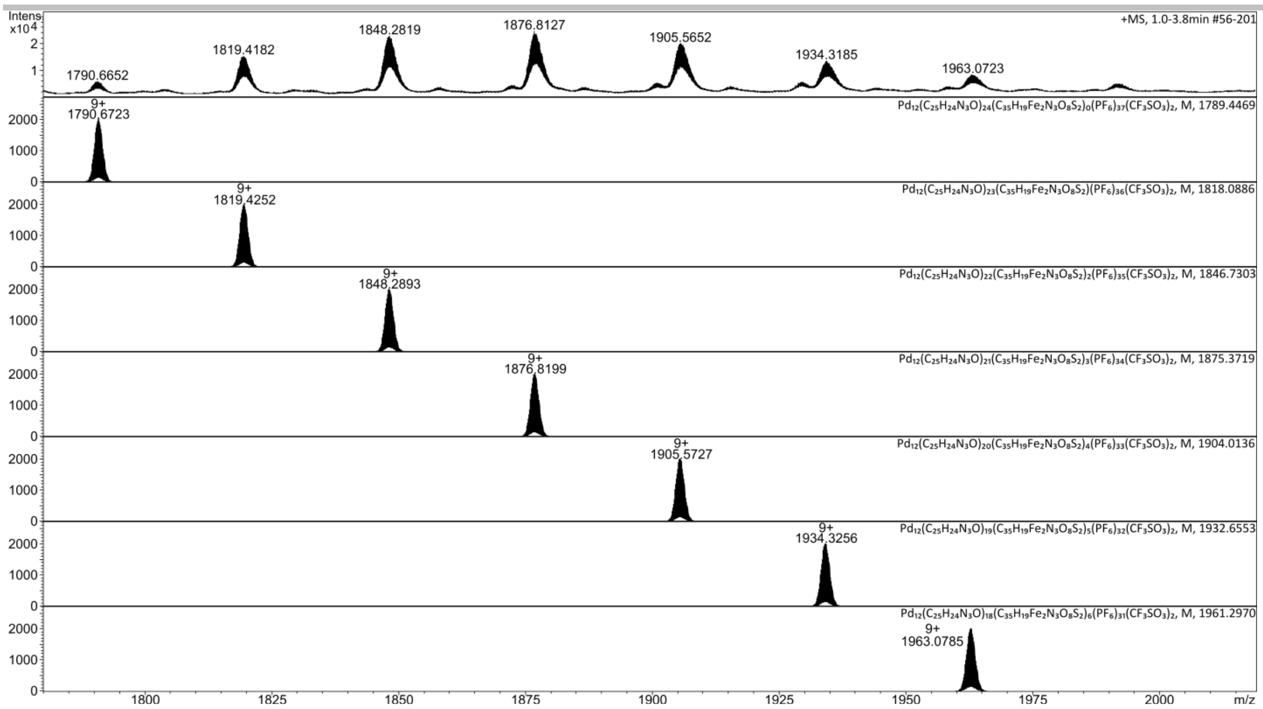

Figure S14. CSI-MS spectra for 9+ species observed for $[Pd_{12}(\mathbf{Fe_2BB})_5(\mathbf{BBNH^+})_{19}]^{43+}$ in CD3CN (top) and calculated species of the type $[Pd_{12}(\mathbf{Fe_2BB})_n(\mathbf{BBNH^+})_{24-n}(PF_6)_x(CF_3SO_3)_2]^{9+}$ (n=0-6; x=31-37). Addition of small amounts of CF_3SO_3H was found to be beneficial for the CSI-MS measurement.

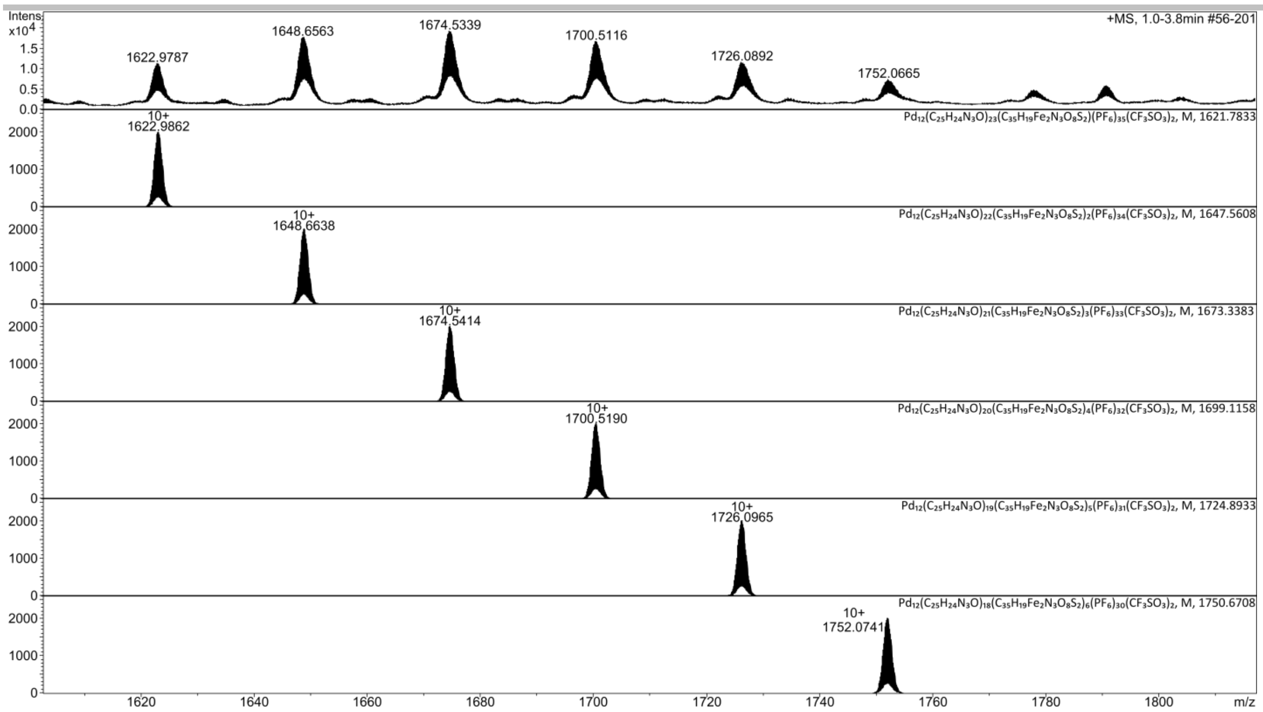

Figure S15. CSI-MS spectra for 10+ species observed for $[Pd_{12}(\mathbf{Fe_2BB})_5(\mathbf{BBNH^+})_{19}]^{43+}$ in CD3CN (top) and calculated species of the type $[Pd_{12}(\mathbf{Fe_2BB})_n(\mathbf{BBNH^+})_{24-n}(PF_6)_x(CF_3SO_3)_2]^{10+}$ (n=1-6; x=30-35). Addition of small amounts of CF_3SO_3H was found to be beneficial for the CSI-MS measurement.

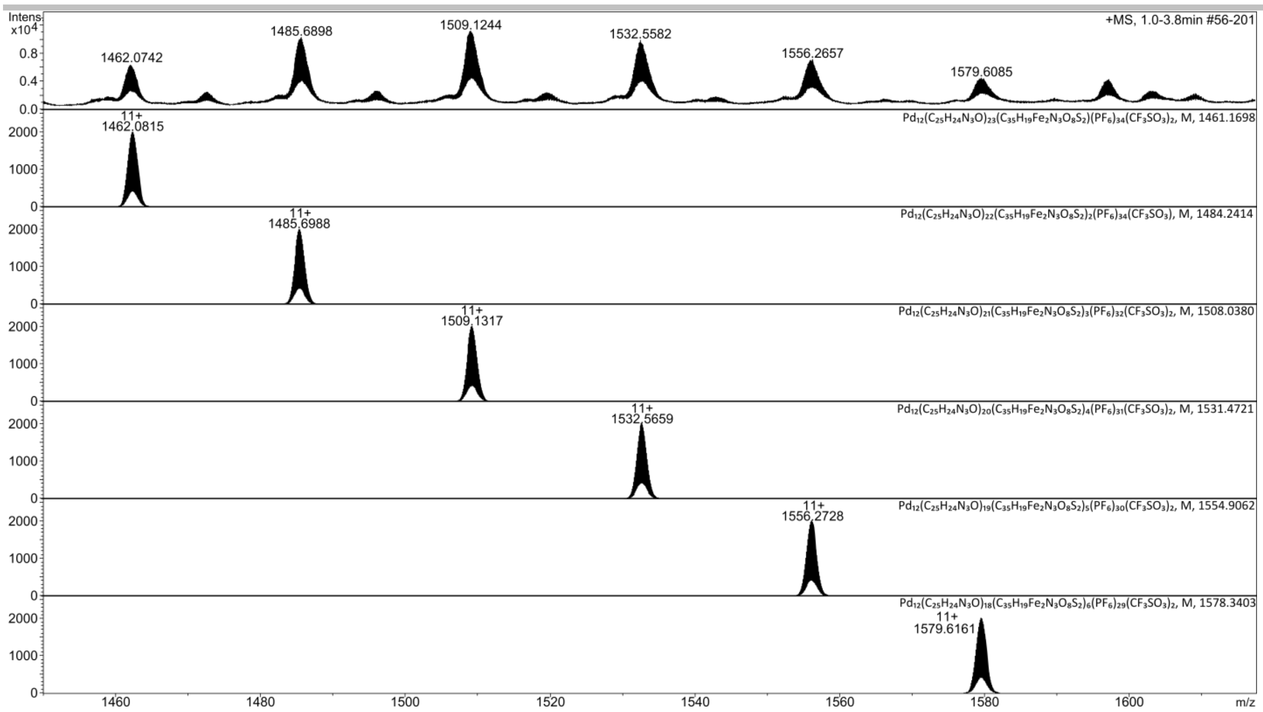

Figure S16. CSI-MS spectra for 11+ species observed for $[Pd_{12}($**Fe$_2$BB**$)_5($**BBNH$^+$**$)_{19}]^{43+}$ in CD3CN (top) and calculated species of the type $[Pd_{12}($**Fe$_2$BB**$)_n($**BBNH$^+$**$)_{24-n}(PF_6)_x(CF_3SO_3)_y]^{11+}$ (n=1-6; x=29-34; y=1,2). Addition of small amounts of CF_3SO_3H was found to be beneficial for the CSI-MS measurement.

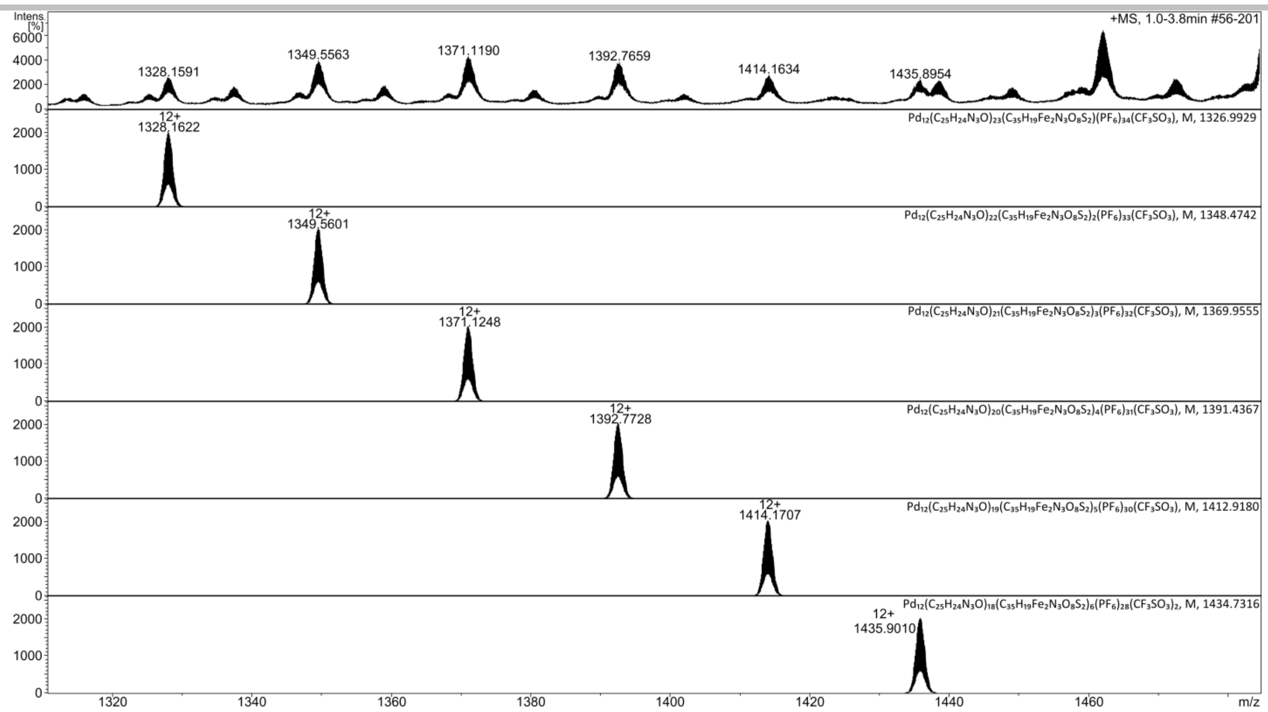

Figure S17. CSI-MS spectra for 12+ species observed for $[Pd_{12}(\mathbf{Fe_2BB})_5(\mathbf{BBNH^+})_{19}]^{43+}$ in CD3CN (top) and calculated species of the type $[Pd_{12}(\mathbf{Fe_2BB})_n(\mathbf{BBNH^+})_{24-n}(PF_6)_x(CF_3SO_3)_y]^{12+}$ (n=1-6; x=28-34, y=1,2). Addition of small amounts of CF_3SO_3H was found to be beneficial for the CSI-MS measurement.

Table S1. Summary of all the species observed with CSI-MS with their respective charge, found m/z and calculated m/z.

Species	Charge	Found [m/z]	Calculated [m/z]
$Pd_{12}(BBNH)_{24}(Fe_2BB)_0(PF_6)_{40}(OTf)_2$	6+	2759.1551	2759.1578
$Pd_{12}(BBNH)_{23}(Fe_2BB)(PF_6)_{39}(OTf)_2$	6+	2802.4511	2802.4539
$Pd_{12}(BBNH)_{22}(Fe_2BB)_2(PF_6)_{38}(OTf)_2$	6+	2844.7499	2844.7495
$Pd_{12}(BBNH)_{21}(Fe_2BB)_3(PF_6)_{37}(OTf)_2$	6+	2887.7156	2887.7122
$Pd_{12}(BBNH)_{24}(Fe_2BB)_0(PF_6)_{39}(OTf)_2$	7+	2343.9943	2343.9973
$Pd_{12}(BBNH)_{23}(Fe_2BB)(PF_6)_{38}(OTf)_2$	7+	2380.8208	2380.8224
$Pd_{12}(BBNH)_{22}(Fe_2BB)_2(PF_6)_{37}(OTf)_2$	7+	2417.9313	2417.9333
$Pd_{12}(BBNH)_{21}(Fe_2BB)_3(PF_6)_{36}(OTf)_2$	7+	2454.4723	2454.4726
$Pd_{12}(BBNH)_{20}(Fe_2BB)_4(PF_6)_{35}(OTf)_2$	7+	2491.2979	2491.2977
$Pd_{12}(BBNH)_{19}(Fe_2BB)_5(PF_6)_{34}(OTf)_2$	7+	2528.2646	2528.2657
$Pd_{12}(BBNH)_{24}(Fe_2BB)_0(PF_6)_{38}(OTf)_2$	8+	2032.8722	2032.8771
$Pd_{12}(BBNH)_{23}(Fe_2BB)(PF_6)_{37}(OTf)_2$	8+	2065.2200	2065.2241
$Pd_{12}(BBNH)_{22}(Fe_2BB)_2(PF_6)_{36}(OTf)_2$	8+	2097.1927	2097.1959
$Pd_{12}(BBNH)_{21}(Fe_2BB)_3(PF_6)_{35}(OTf)_2$	8+	2129.6647	2129.6680
$Pd_{12}(BBNH)_{20}(Fe_2BB)_4(PF_6)_{34}(OTf)_2$	8+	2161.7618	2161.7649
$Pd_{12}(BBNH)_{19}(Fe_2BB)_5(PF_6)_{33}(OTf)_2$	8+	2193.9844	2193.9868
$Pd_{12}(BBNH)_{24}(Fe_2BB)_0(PF_6)_{37}(OTf)_2$	9+	1790.6652	1790.6723
$Pd_{12}(BBNH)_{23}(Fe_2BB)(PF_6)_{36}(OTf)_2$	9+	1819.4182	1819.4252
$Pd_{12}(BBNH)_{22}(Fe_2BB)_2(PF_6)_{35}(OTf)_2$	9+	1848.2819	1848.2893
$Pd_{12}(BBNH)_{21}(Fe_2BB)_3(PF_6)_{34}(OTf)_2$	9+	1876.8127	1876.8199
$Pd_{12}(BBNH)_{20}(Fe_2BB)_4(PF_6)_{33}(OTf)_2$	9+	1905.5652	1905.5727
$Pd_{12}(BBNH)_{19}(Fe_2BB)_5(PF_6)_{32}(OTf)_2$	9+	1934.3185	1934.3256
$Pd_{12}(BBNH)_{18}(Fe_2BB)_6(PF_6)_{31}(OTf)_2$	9+	1963.0723	1963.0785
$Pd_{12}(BBNH)_{23}(Fe_2BB)(PF_6)_{35}(OTf)_2$	10+	1622.9787	1622.9862
$Pd_{12}(BBNH)_{22}(Fe_2BB)_2(PF_6)_{34}(OTf)_2$	10+	1648.6563	1648.6638
$Pd_{12}(BBNH)_{21}(Fe_2BB)_3(PF_6)_{33}(OTf)_2$	10+	1674.5339	1674.5414
$Pd_{12}(BBNH)_{20}(Fe_2BB)_4(PF_6)_{32}(OTf)_2$	10+	1700.5116	1700.5190
$Pd_{12}(BBNH)_{19}(Fe_2BB)_5(PF_6)_{31}(OTf)_2$	10+	1726.0892	1726.0965
$Pd_{12}(BBNH)_{18}(Fe_2BB)_6(PF_6)_{30}(OTf)_2$	10+	1752.0665	1752.0741
$Pd_{12}(BBNH)_{23}(Fe_2BB)(PF_6)_{34}(OTf)_2$	11+	1462.0742	1462.0815
$Pd_{12}(BBNH)_{22}(Fe_2BB)_2(PF_6)_{34}(OTf)$	11+	1485.6898	1485.6988
$Pd_{12}(BBNH)_{21}(Fe_2BB)_3(PF_6)_{32}(OTf)_2$	11+	1509.1244	1509.1317
$Pd_{12}(BBNH)_{20}(Fe_2BB)_4(PF_6)_{31}(OTf)_2$	11+	1532.5582	1532.5659
$Pd_{12}(BBNH)_{19}(Fe_2BB)_5(PF_6)_{30}(OTf)_2$	11+	1556.2657	1556.2728
$Pd_{12}(BBNH)_{18}(Fe_2BB)_6(PF_6)_{29}(OTf)_2$	11+	1579.6085	1579.6161
$Pd_{12}(BBNH)_{23}(Fe_2BB)(PF_6)_{34}(OTf)$	12+	1328.1591	1328.1622
$Pd_{12}(BBNH)_{22}(Fe_2BB)_2(PF_6)_{33}(OTf)$	12+	1349.5563	1349.5601
$Pd_{12}(BBNH)_{21}(Fe_2BB)_3(PF_6)_{32}(OTf)$	12+	1371.1190	1371.1248
$Pd_{12}(BBNH)_{20}(Fe_2BB)_4(PF_6)_{31}(OTf)$	12+	1392.7659	1392.7728
$Pd_{12}(BBNH)_{19}(Fe_2BB)_5(PF_6)_{30}(OTf)$	12+	1414.1634	1414.1707
$Pd_{12}(BBNH)_{18}(Fe_2BB)_6(PF_6)_{28}(OTf)_2$	12+	1435.8954	1435.9010

Cage[$Pd_{12}(Fe_2BB)_5(BB)_{19}]^{24+}$

Preparation: A Schlenk flask was charged with 3.93 mg (5 equiv, 5 µmol) of **Fe_2BB**, 5.33 mg (19eq, 19µmol) of **BB** and 6.72 mg (12 equiv, 12 µmol) of [$Pd(PF_6)_2(MeCN)_4$]. The flask was flushed with nitrogen before 5 ml of degassed CD_3CN were added. The resulting mixture was heated under N_2 at 60 °C overnight.

NMR Spectroscopy

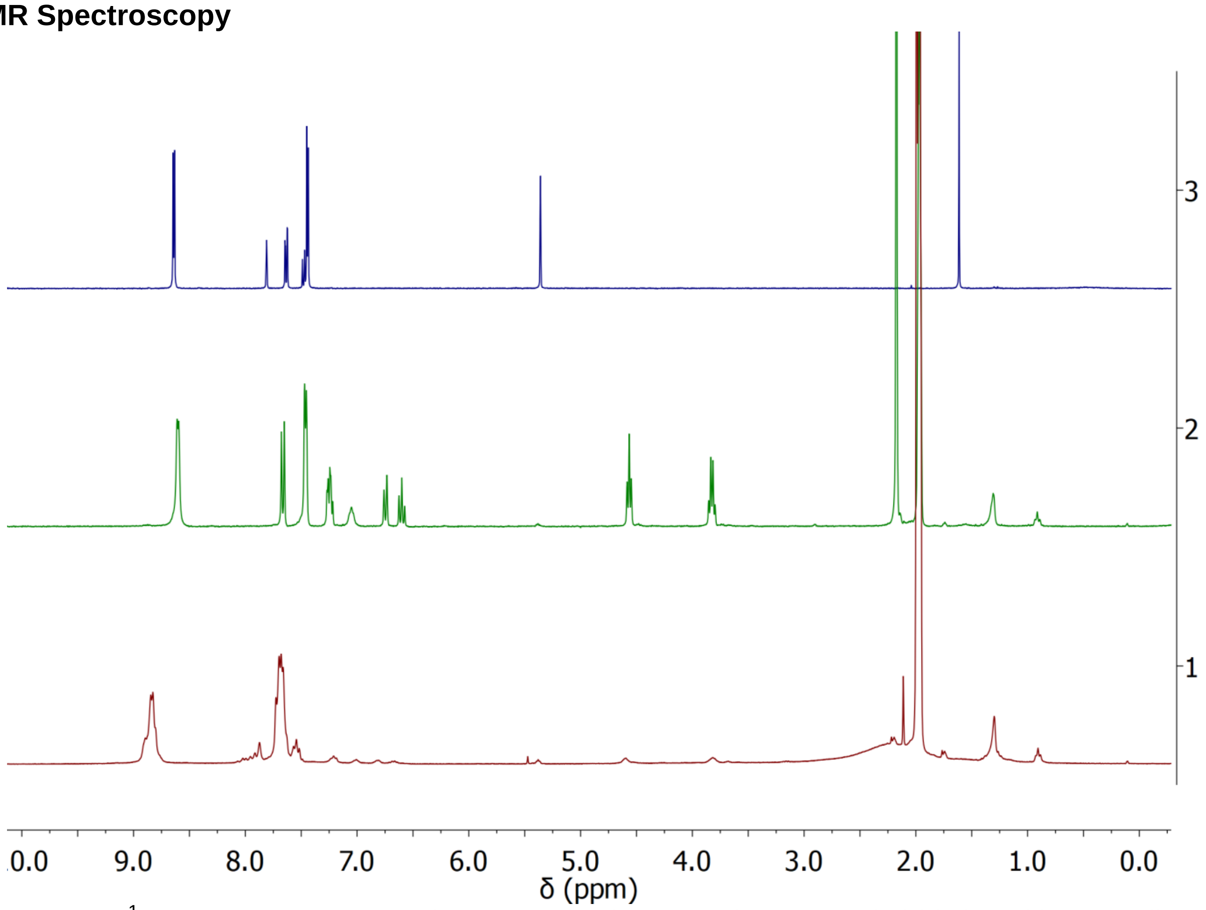

Figure S18. ^{1}H-NMR spectra of **BB** in CD_2Cl_2 (top), **Fe_2BB** in CD_3CN (middle) and its palladium cage [Pd_{12}(**Fe_2BB**)$_5$(**BB**)$_{19}$]$^{43+}$ in CD_3CN (bottom). Note shift of the pyridine peaks upon metal coordination.

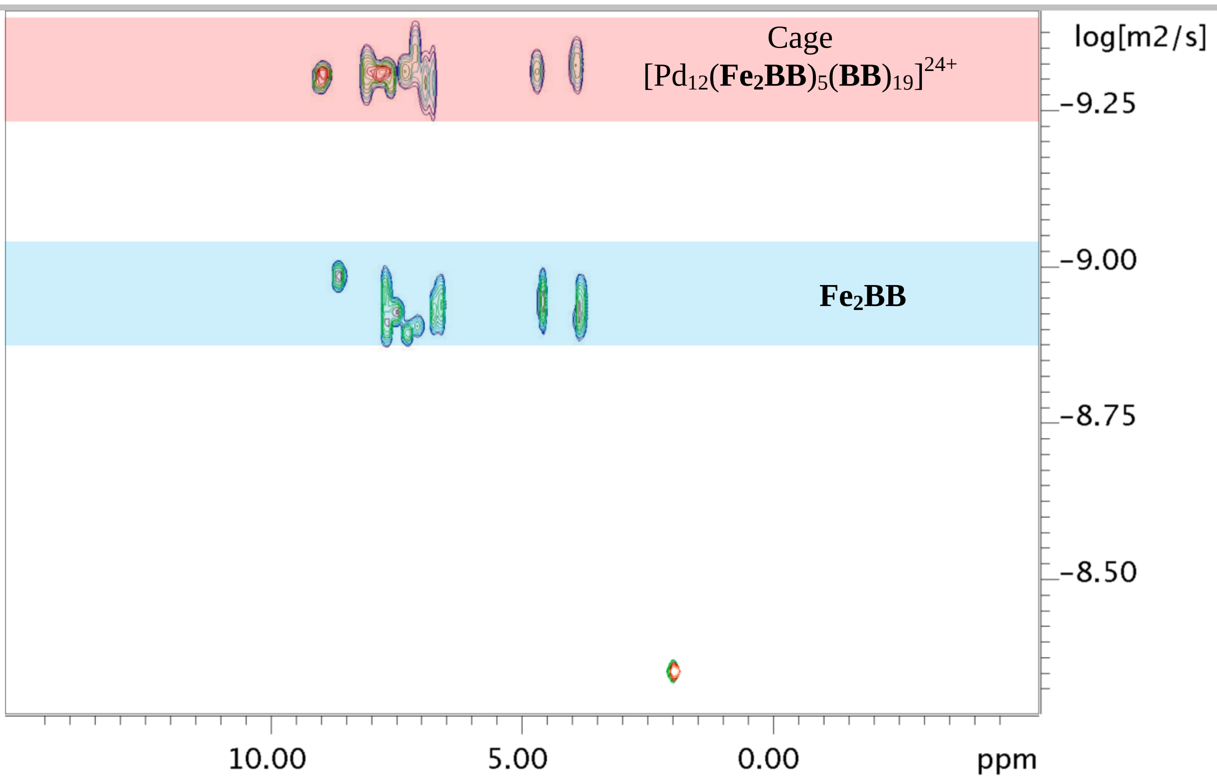

Figure S19. Overlay of ^{1}H DOSY NMR in MeCN-*d3* at 25 °C for a mixed cage of the type [Pd_{12}(**Fe_2BB**)$_5$(**BB**)$_{19}$]$^{24+}$ with logD of -9.3 m^2s^{-1} (top). This diffusing species contains the signals for both the unfunctionalized building block **BB** and for the di-iron functionalized building block. The **Fe_2BB** shows a logD value of -8.9 m^2s^{-1} (bottom).

Electrochemistry

Fe_2BB

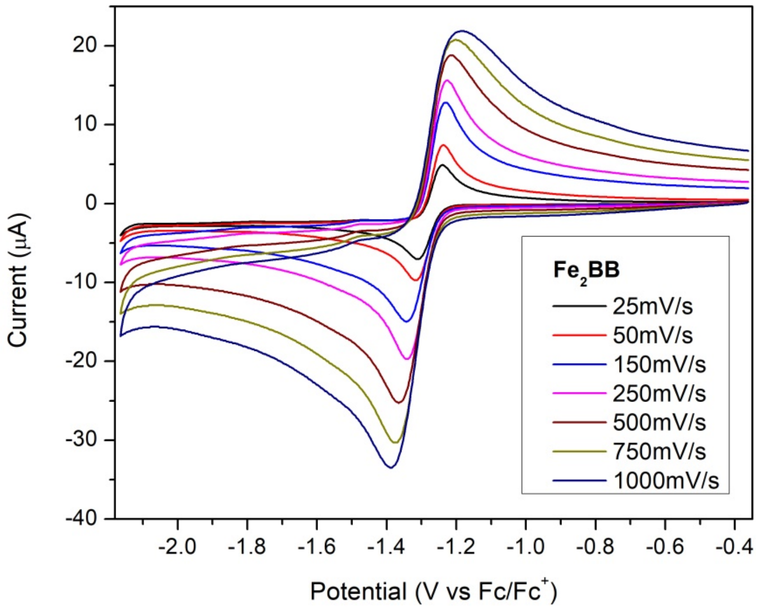

Figure S20. Cyclic voltammograms recorded at different scan speeds for a solution of **Fe_2BB** in MeCN.

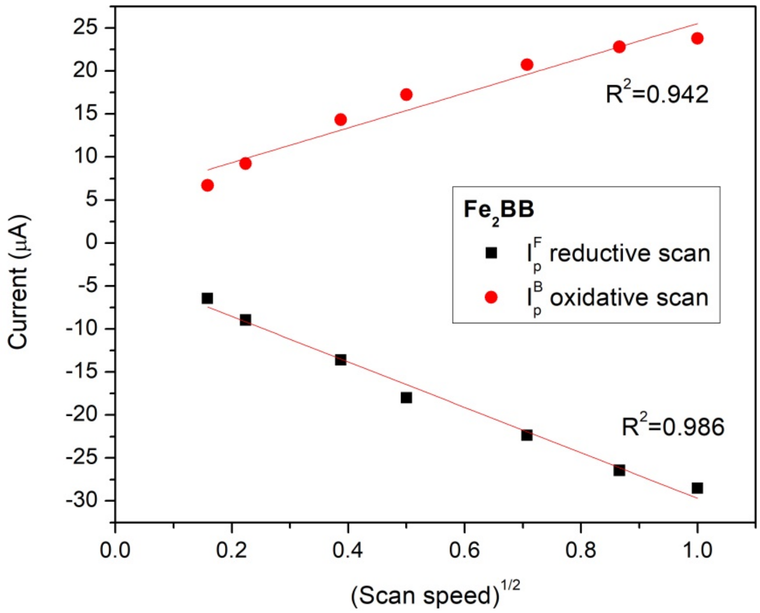

Figure S21. Plot of peak current vs. square root of the scan speed for a solution **Fe_2BB** in MeCN.

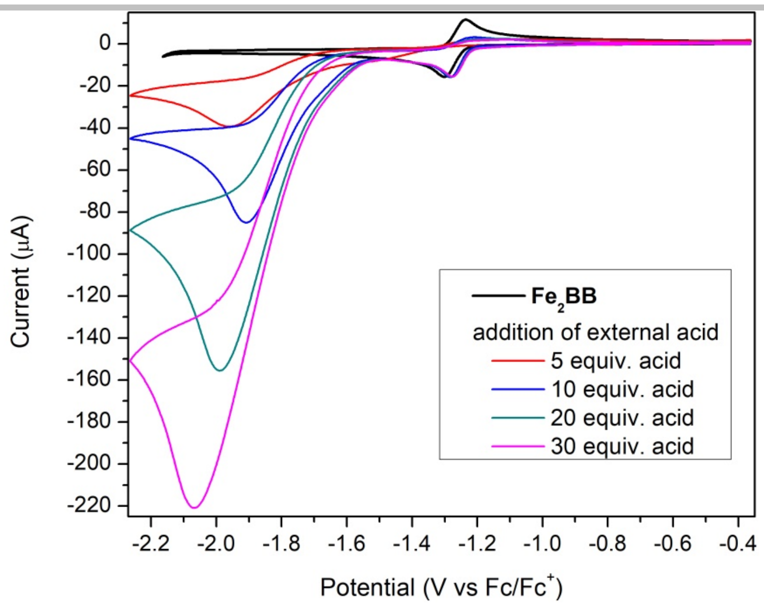

Figure S22. Cyclic voltammograms for a solution of **Fe_2BB** in MeCN. with addition of different amounts of external acid ($HNEt_3PF_6$) showing catalytic proton reduction at around -1.05V vs Fc/Fc^+.

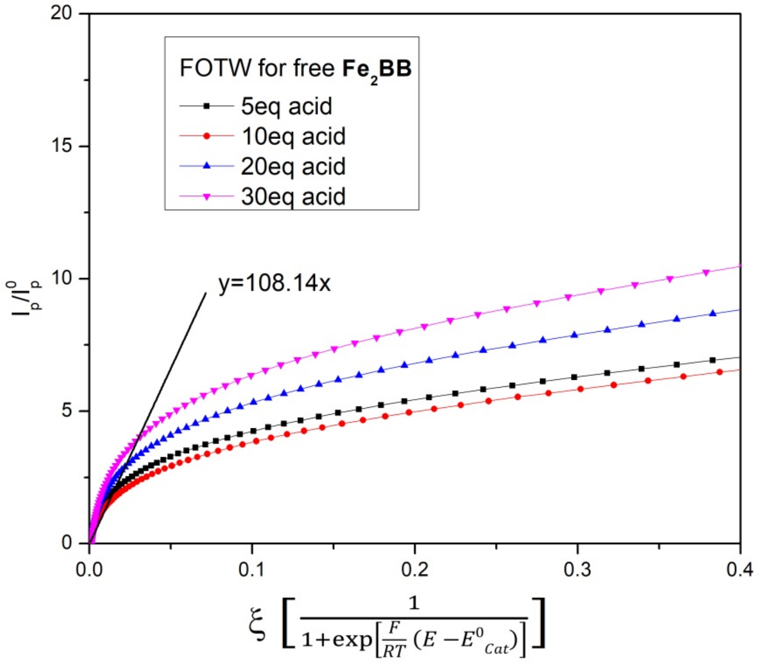

Figure S23. Foot of the wave analysis (FOTW) for **Fe_2BB** in MeCN, (1mM, scan speed 100mV/s) in the presence of different equivalents of external acid ($HNEt_3PF_6$). The slope reported in the graph was used to calculate k_{cat} according to Equation S1. For a 30mM concentration of substrate k_{cat} is calculated to be $1.51 \cdot 10^5$ $mol^{-1}s^{-1}$ and TOF_{max} is calculated via Equation S2 to be $9.06 \cdot 10^3$ s^{-1}.

$$slope = 2.24\sqrt{\frac{RT}{Fv}2k_{cat}[H^+]}$$

Equation S1. Expression to calculate k_{cat} from the slope obtained from FOTW. $[H^+]$ is the initial acid concentration while R is gas constant, T is temperature, F is Faraday constant and v is the scan speed.

$$TOF_{max} = 2k_{cat}[H^+]$$

Equation S2. Expression to calculate TOF_{max}. $[H^+]$ is the initial acid concentration.

Cage [Pd$_{12}$(Fe$_2$BB)$_5$(BB)$_{19}$]$^{24+}$

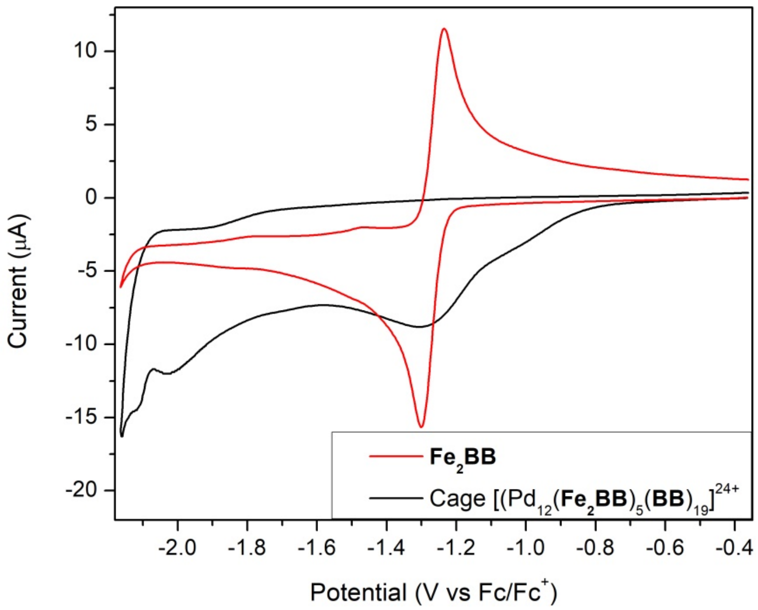

Figure S24. Cyclic voltammograms for a solution of cage [Pd$_{12}$(**Fe$_2$BB**)$_5$(**BB**)$_{19}$]$^{24+}$ in MeCN. (black) and free **Fe$_2$BB** (red) in the absence of external acid to compare the reduction of the diiron catalyst at about -1.3 V vs Fc/Fc$^+$. In clear contrast with free **Fe$_2$BB** the voltammogram for the cage system it is not reversible but it is noted that if potential window -1.9 to -2.2 V is entered cage decomosition occours and the working electrode must be cleaned and polished before subsequent measurement.
This decomposition and deposition on the working electrode is not observed when protons are present in solution as showed in Figure S25.

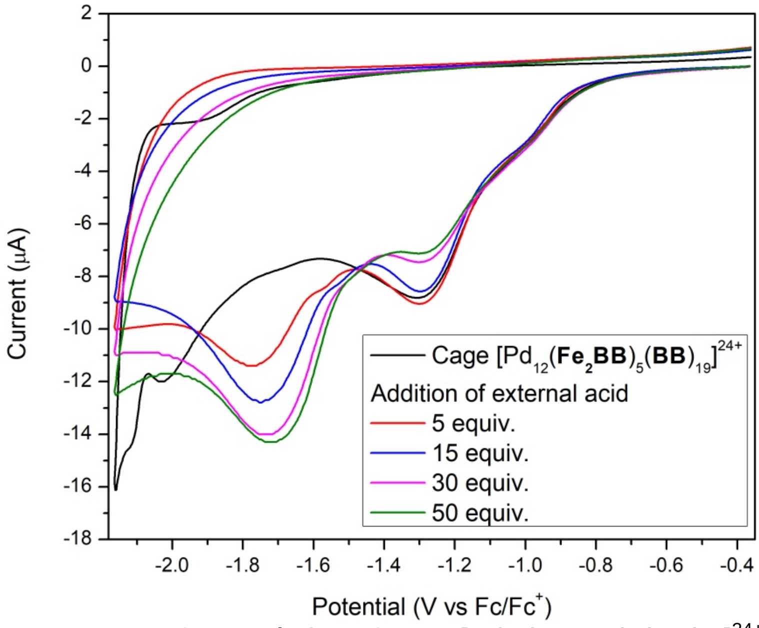

Figure S25. Cyclic voltammograms for a solution of cage [Pd$_{12}$(**Fe$_2$BB**)$_5$(**BB**)$_{19}$]$^{24+}$ in MeCN. without (black) and with addition of different amounts of external acid (HNEt$_3$PF$_6$) showing catalytic proton reduction at around -1.7 V vs Fc/Fc$^+$.

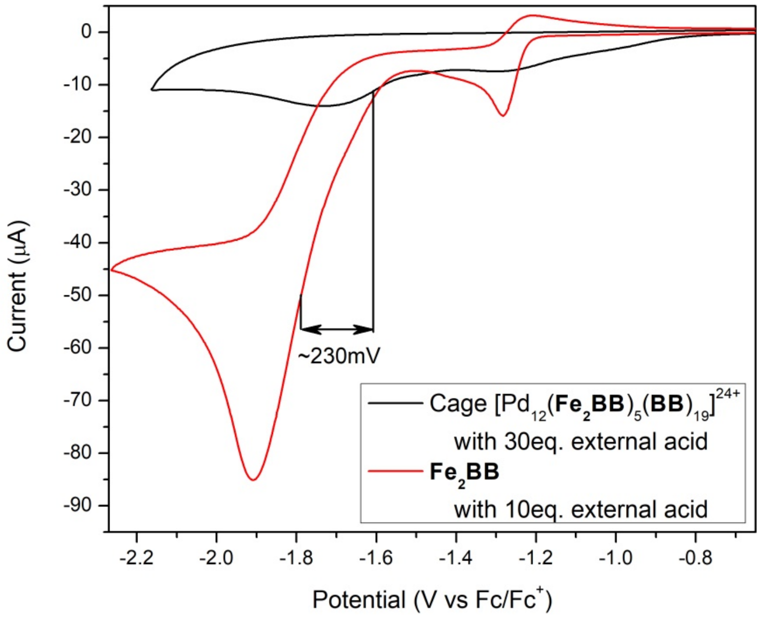

Figure S26. Cyclic voltammograms for a solution of cage $[Pd_{12}(\mathbf{Fe_2BB})_5(\mathbf{BB})_{19}]^{24+}$ in MeCN with 30 equivalents of external acid (black) and free $\mathbf{Fe_2BB}$ in with 10 equivalents of external acid. For the cage system, proton reduction takes place at about 230 mV milder potential.

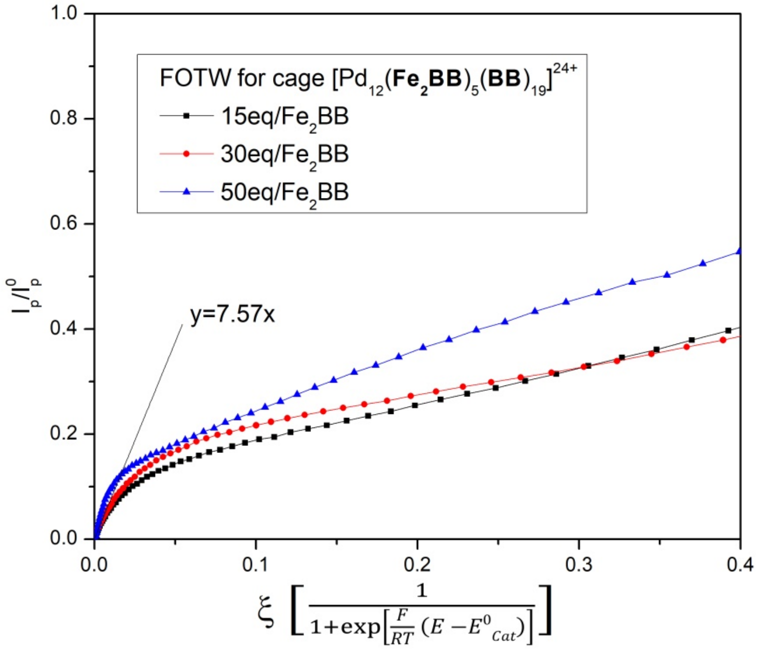

Figure S27. Foot of the wave analysis (FOTW) for cage $[Pd_{12}(\mathbf{Fe_2BB})_5(\mathbf{BB})_{19}]^{24+}$ in MeCN, (cage 0.2mM, scan speed 100mV/s) in the presence of different equivalents of external acid ($HNEt_3PF_6$). The slope reported in the graph was used to calculate k_{cat} according to Equation S1. For a 30mM concentration of substrate k_{cat} is calculated to be $7.42 \cdot 10^2$ mol^{-1}s^{-1} and TOF_{max} is calculated via Equation S2 to be $4.45 \cdot 10^1$ s^{-1}.

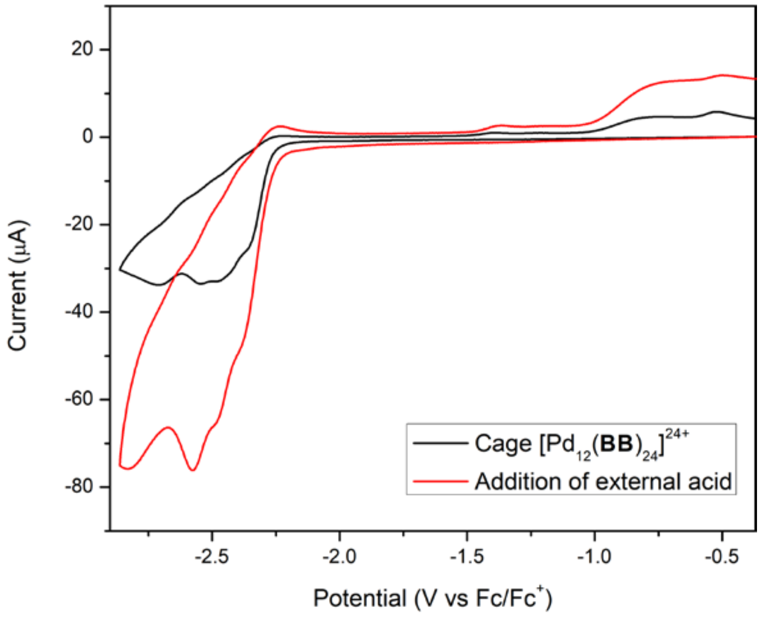

Figure S28. Cyclic voltammograms for a solution of cage $[Pd_{12}(\mathbf{BB})_{24}]^{24+}$ in MeCN (black) and with the addtition of 10 equivalents of external acid (red). The cage $[Pd_{12}(\mathbf{BB})_{24}]^{24+}$ does not containg functional groups or diiron catalyst. This experiments provides insignts into cage stability under electrochemical conditions with and without external acid present.

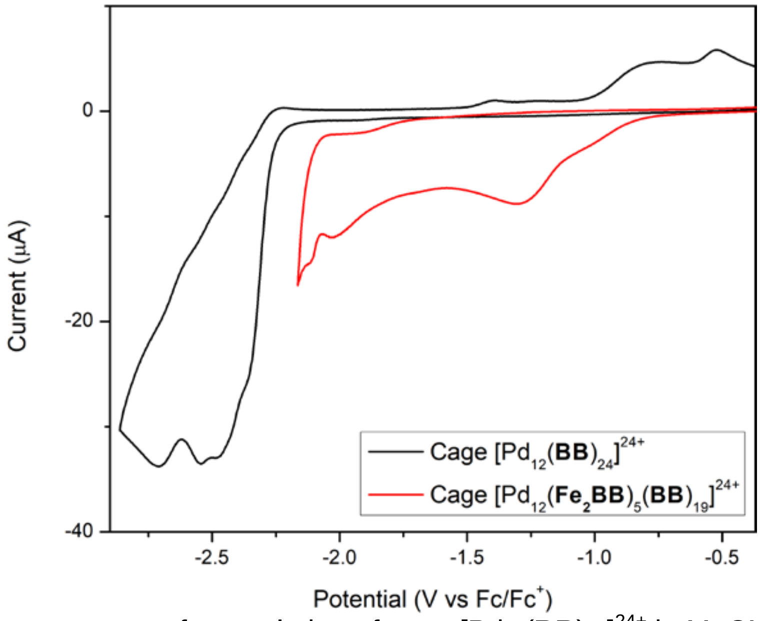

Figure S29. Cyclic voltammograms for a solution of cage $[Pd_{12}(\mathbf{BB})_{24}]^{24+}$ in MeCN (black) and cage $[Pd_{12}(\mathbf{Fe_2BB})_5(\mathbf{BB})_{19}]^{24+}$ in MeCN (red) as further confirmation that the peak around -1.25 V belongs to the reductiond of the encapsulated diiron catalyst

Cage [Pd$_{12}$(Fe$_2$BB)$_5$(BBNH$^+$)$_{19}$]$^{43+}$

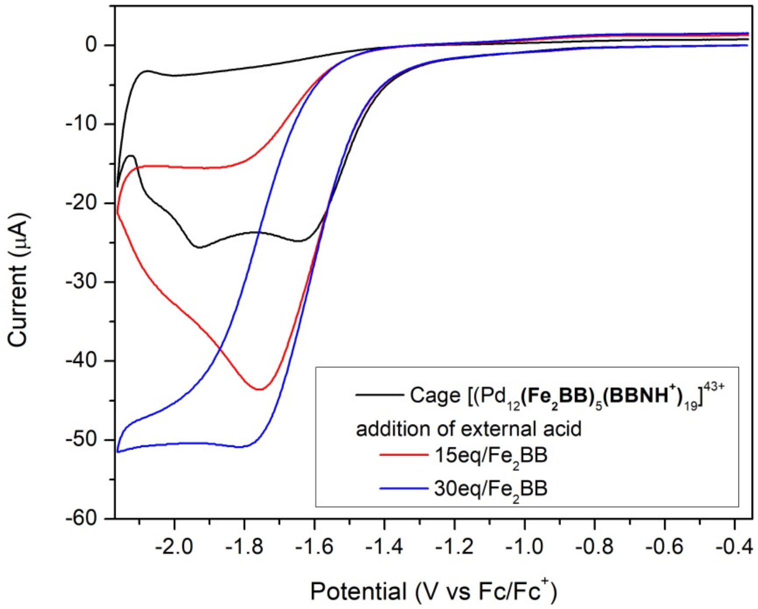

Figure S30. Cyclic voltammograms for a solution of cage [Pd$_{12}$(**Fe$_2$BB**)$_5$(**BBNH$^+$**)$_{19}$]$^{43+}$ in MeCN. without (black) and with addition of different amounts of external acid (HNEt$_3$PF$_6$) (red and blue) showing catalytic proton reduction at around -1.7 V vs Fc/Fc$^+$.

The [Pd$_{12}$(**Fe$_2$BB**)$_5$(**BBNH$^+$**)$_{19}$]$^{43+}$ cage can be prepared in a 0.2 mM concentration but it is noted that when electrolyte (TBAPF$_6$) is added to this solution, copious amount of precipitate appears. Therefore, to avoid such undesired event, the cage solution is diluted ten times, thereby yielding a concentration of the encapsulated di-iron moiety of about 0.1 mM. The expected peak current for the reduction of the di-iron complex, predicted by Randles–Sevcik equation, is only roughly -1.6 µA, given the di-iron concentration and cage diffusion coefficient obtained from ^{1}H DOSY NMR. Such small current cannot be clearly distinguished in the voltammogram obtained. As the di-iron complex is present in such low concentration that should give first reduction current of -1.6 µA at -1.3 V *vs* Fc$^{0/+}$, the reduction event observed at -1.7 V *vs* Fc$^{0/+}$ with a peak current of -25 µA suggests a catalytic reduction event.

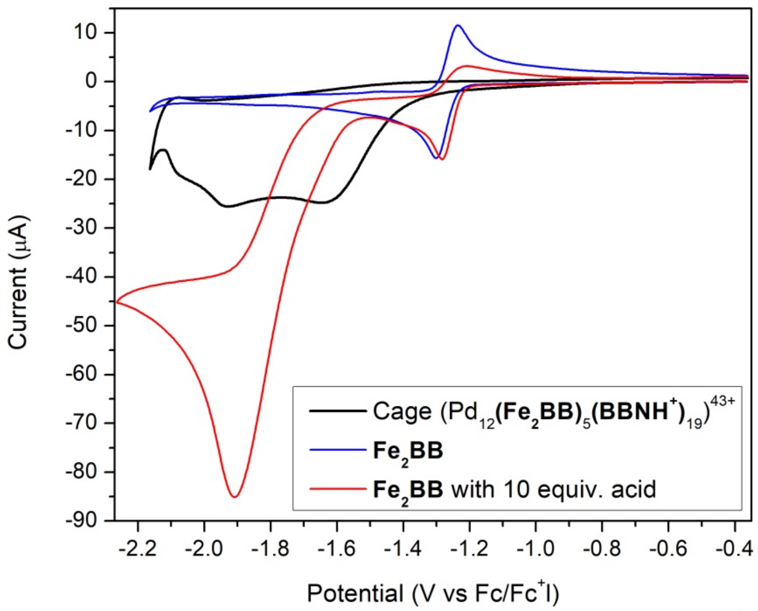

Figure S31. Cyclic voltammograms for a solution of cage [Pd_{12}(**Fe_2BB**)$_5$(**BBNH$^+$**)$_{19}$]$^{43+}$ in MeCN. without addition of external acid (black) and free **Fe_2BB** without addition of external acid (blue) and with 10 equivalents of external acid (red). For the cage system, proton reduction takes place at about 250 mV milder potential.

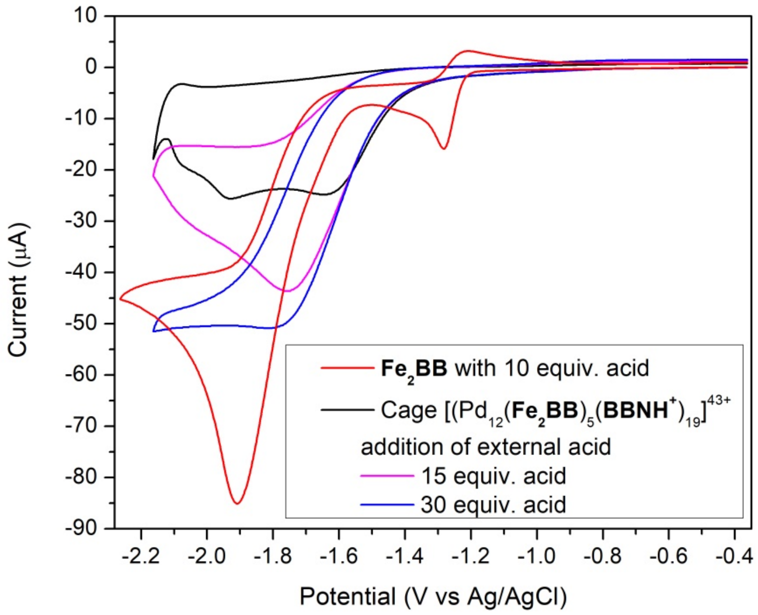

Figure S32. Cyclic voltammograms for a solution of cage [Pd_{12}(**Fe_2BB**)$_5$(**BBNH$^+$**)$_{19}$]$^{43+}$ in MeCN. without addition of external acid (black), with 15 and 30 equivalents of external acid (pink and blue) and free **Fe_2BB** with 10 equivalents of external acid (red). For the cage system, proton reduction takes place at about 250 mV milder potential.

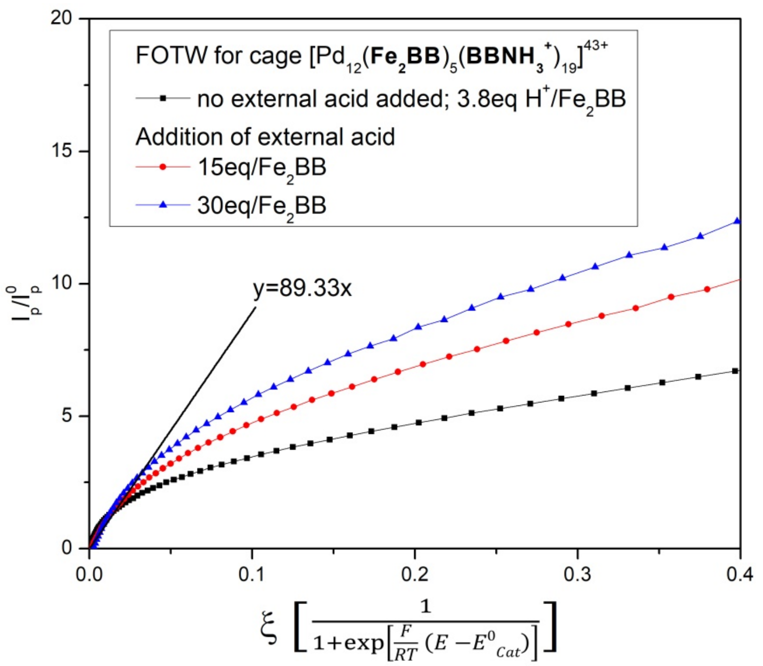

Figure S33. Foot of the wave analysis (FOTW) for cage $[Pd_{12}($**Fe_2BB**$)_5($**$BBNH^+$**$)_{19}]^{43+}$ in MeCN, (cage 0.02mM, scan speed 100mV/s) in the presence of different equivalents of external acid ($HNEt_3PF_6$). The slope reported in the graph was used to calculate k_{cat} according to Equation S1. For a 30mM concentration of substrate k_{cat} is calculated to be $1.03{\cdot}10^5$ $mol^{-1}s^{-1}$ and TOF_{max} is calculated via Equation S2 to be $6.20{\cdot}10^3$ s^{-1}.

Spectroelectrochemistry

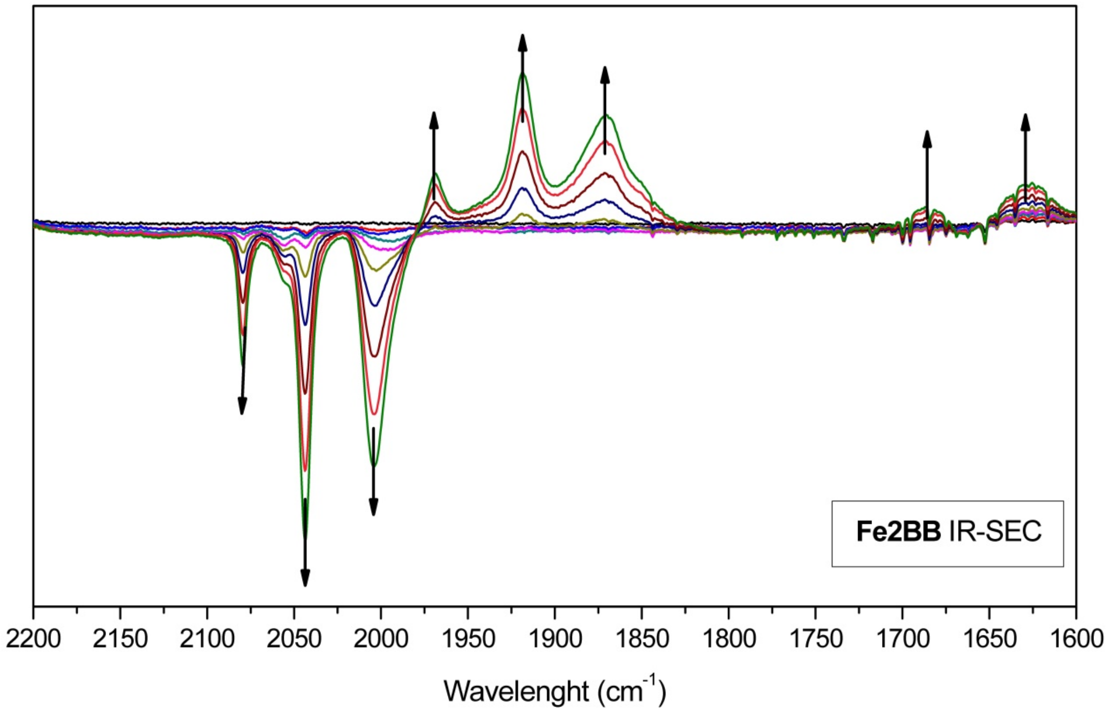

Figure S34. IR-coupled spectroelectrochemical measurement for **Fe_2BB** in MeCN showing the bleaching of carbonyl peaks of the neutral complex (negative part) and the appearance of the doubly reduced species (positive part).

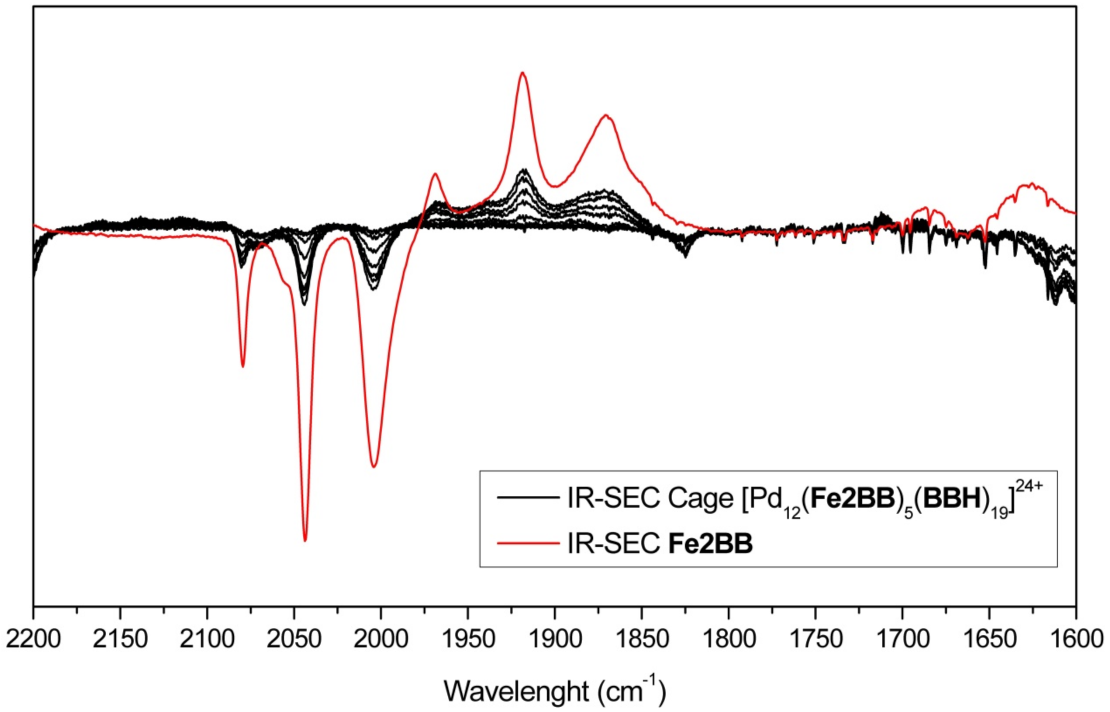

Figure S35. IR-coupled spectroelectrochemical measurement for cage $[Pd_{12}(\mathbf{Fe_2BB})_5(\mathbf{BB})_{19}]^{24+}$ (black) showing the bleaching of carbonyl peaks of the neutral complex (negative part) and the appearance of the doubly reduced species (positive part) and overlap with spectroelectrochemical measurement for **Fe_2BB** (red) to show that **Fe_2BB** can be electrochemically accessed and reduced at the same potential (~-1.3 V) even when part of the large assembly. It is noted that if potential window -1.9 to -2.2 V is entered cage decomposition occours (see Figure S24).

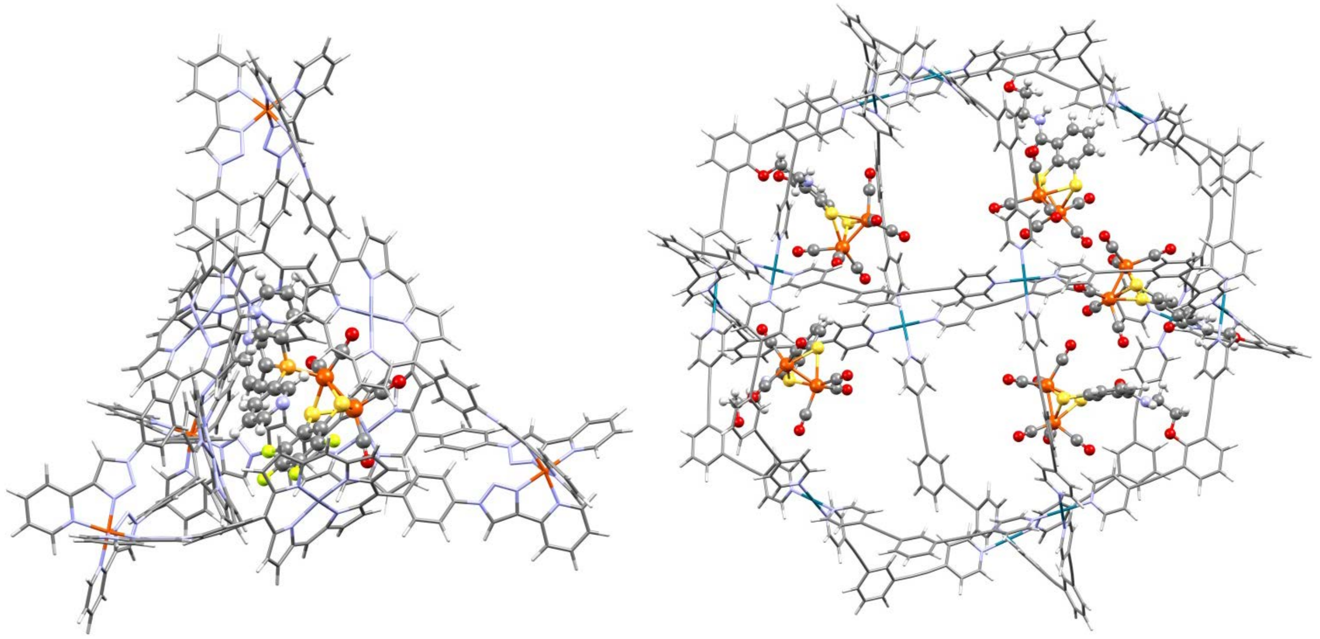

Figure S36. Left: structure of cage {[$Fe_4(ZnL)_6$][$Fe_2(F_4bdt)(PPy_3)(CO_5)$]}$^{8+}$ showing a tight fit of the encapsulated catalyst, its proximity to cage walls and exposure to bulk solvent and substrate.[6] Functional groups on porphyrin rings (Me and Et groups) are omitted for clarity. Right: structure of the larger cage [Pd_{12}(**Fe_2BB**)$_5$(**BB**)$_{19}$]$^{24+}$ showing simultaneous encapsulation of several catalysts and their increased distance to cage walls compared to the smaller cage on the left. Structures are optimized at molecular mechanics level (MMFF) and shown in wire-style; carbon in grey, hydrogen in white, nitrogen in cyan, oxygen in red, iron cage corners in orange and palladium cage corners in blue. The di-iron catalysts are represented in ball and stick style; iron in orange, sulfur in yellow, carbon in gray, oxygen in red, nitrogen in cyan, phosphorus in light orange and fluorine in green.

References

[1] *Bruker, APEX2 software, Madison WI, USA, 2014*.
[2] S. G. M. Sheldrick, Universität Göttingen, Germany, 2008.
[3] *G. M. Sheldrick, SHELXL2013, University of Göttingen, Germany, 2013.*
[4] R. Zaffaroni, E. O. Bobylev, R. Plessius, J. I. van der Vlugt, J. N. H. Reek, *J. Am. Chem. Soc.* **2020**, *142*, 8837-8847.
[5] R. Zaffaroni, R. J. Detz, J. I. van der Vlugt, J. N. H. Reek, *ChemSusChem* **2018**, *11*, 209-218.
[6] S. S. Nurttila, R. Zaffaroni, S. Mathew, J. N. H. Reek, *Chem. Commun.* **2019**, *55*, 3081-3084.
